# *Nannochloropsis oceanica* IMET1 and its bacterial symbionts for carbon capture, utilization, and storage: biomass and calcium carbonate production under high pH and high alkalinity

**DOI:** 10.1128/aem.00133-25

**Published:** 2025-04-17

**Authors:** Lauren Jonas, Yi-Ying Lee, Robert Mroz, Russell T. Hill, Yantao Li

**Affiliations:** 1 University of Maryland Center for Environmental Science 12265, Baltimore, Maryland, USA; 2 Institute of Marine and Environmental Technology 145781 , Baltimore, Maryland, USA; 3 HY-TEK Bio, LLC, Baltimore, Maryland, USA; University of Delaware, Lewes, Delaware, USA

**Keywords:** microalgae, bacterial symbionts, carbon capture, utilization, and storage (CCUS), sodium bicarbonate, MICP, calcium carbonate, Patescibacteria

## Abstract

To combat the increasing levels of carbon dioxide (CO_2_) released from the combustion of fossil fuels, microalgae have emerged as a promising strategy for biological carbon capture, utilization, and storage. This study used a marine microalgal strain, *Nannochloropsis oceanica* IMET1, which thrives in high CO_2_ concentrations. A high-pH, high-alkalinity culture was designed for CO_2_ capture through algal biomass production as well as permanent sequestration through calcium carbonate (CaCO_3_) precipitation. This was accomplished by timed pH elevation and the addition of sodium bicarbonate to cultures of *N. oceanica* grown at lab scale (1 L) and pilot scale (500 L) with 10% and 5% CO_2_, respectively. Our data showed that 0.02 M NaHCO_3_ promoted algal growth and that sparging cultures with ambient air after 12 days raised pH and created favorable CaCO_3_ formation conditions. At the 1 L scale, we reached 1.52 g L^−1^ biomass after 12 days and an extra 9.3% CO_2_ was captured in the form of CaCO_3_ precipitates. At the 500 L pilot scale, an extra 60% CO_2_ was captured (Day 40) with a maximum CO_2_ capture rate of 63.2 g m^−2^ day^−1^ (Day 35). Bacterial communities associated with the microalgae were dominated by two novel Patescibacteria. Functional analysis revealed that genes for several plant growth-promotion traits (PGPTs) were enriched within this group. The microalgal-bacterial coculture system offers advantages for enhanced carbon mitigation through biomass production and simultaneous precipitation of recalcitrant CaCO_3_ for long-term CO_2_ storage.

**IMPORTANCE:**

Capturing carbon dioxide (CO_2_) released from fossil fuel combustion is of the utmost importance as the impacts of climate change continue to worsen. Microalgae can remove CO_2_ through their natural photosynthetic pathways and are additionally able to convert CO_2_ into a stable, recalcitrant form as calcium carbonate (CaCO_3_). We demonstrate that microalgae-based carbon capture systems can be greatly improved with high pH and high alkalinity by providing optimal conditions for carbonate precipitation. Our results with the microalga, *Nannochloropsis oceanica* strain IMET1, show an extra 9.3% CO_2_ captured as CaCO_3_ at the 1 L scale and an extra 60% CO_2_ captured at the 500 L (pilot) scale. Our optimized system provides a novel approach to capture CO_2_ through two mechanisms: (i) as organic carbon within microalgal biomass and (ii) as inorganic carbon stored permanently in the form of CaCO_3._

## INTRODUCTION

Dependence on fossil fuels for energy has elevated the levels of carbon dioxide (CO_2_) in the atmosphere, resulting in deleterious effects on the Earth’s biosphere (1, 2). Developing strategies to capture and store this excess atmospheric CO_2_ is one of mankind’s greatest challenges (3). Promisingly, microalgae can remove and sequester CO_2_ by way of their photosynthetic pathway (4). Captured CO_2_ in microalgal biomass can be utilized for the production of biofuel and bioproducts such as antioxidants and aquaculture feed (5). CO_2_ can additionally be precipitated and permanently stored in an inorganic form, such as calcium carbonate (CaCO_3_) (6).

Coal-fired power plants typically produce flue gas with 12%–15% CO_2_ by volume while natural gas-fired power plants produce 4%–5% (as provided by the National Energy Technology Laboratory, https://www.netl.doe.gov/carbon-capture/power-generation). Many microalgae can grow only under atmospheric CO_2_ concentrations of approx. 0.042%. However, a few species have been found to thrive under elevated CO_2_ conditions (7). This study used a strain in the marine *Nannochloropsis* genus, members of which show improved growth with high CO_2_ concentrations (≥15%) (8, 9) and with simulated flue gas (10). We chose *N. oceanica* IMET1 because of its ability to grow rapidly under high CO_2_ concentrations, its high content of lipids including triacylglycerols (TAGs) and high-value omega-3 fatty acids such as eicosapentaenoic acid (EPA), its production of carotenoids (11, 12), its well-characterized genome (13), and its established genetic modification methods (14).

Recent studies show that sodium bicarbonate (NaHCO_3_) can enhance the biomass and biochemical content of microalgae, including *Nannochloropsis* sp (15, 16). NaHCO_3_ has been used as an inorganic carbon replacement for CO_2_ gas, but few studies have considered the potential of the synergistic effects of both carbon sources. The addition of NaHCO_3_ creates a high-pH, high-alkalinity culture that is ideal for microalgal growth and can enhance CO_2_ capture as CaCO_3_ through Microbially Induced Carbonate Precipitation (MICP) (17). MICP facilitated by photosynthesis occurs frequently in nature with microalgae and cyanobacteria being the main microorganisms responsible for MICP in aquatic environments (6). This phenomenon has been observed at a laboratory scale and is governed by the following four main factors: calcium availability, DIC concentration, pH, and nucleation site for precipitation (18, 19). The ability of microalgae to capture and store atmospheric carbon as recalcitrant CaCO_3_ is a strategy that remains largely unexploited.

An often-overlooked factor in applied phycology is the significance of symbiotic associations between microalgae and bacteria, including the wide range of benefits that bacterial cells can provide to their hosts (20). The main mechanisms by which bacterial communities can enhance microalgal growth are by excreting phytohormones and vitamins (21, 22). Bacteria are also major players in MICP processes. A recent study found that microalgal/bacterial cocultures promoted CaCO_3_ formation through MICP in the marine environment (23).

We previously designed a prototype system for microalgae-driven calcium carbonate and biomass production (MadCAP) to grow algae and capture carbon with ambient air (24). This paper aimed to test the MadCAP system using 5%–10% CO_2_ (simulated power plant flue gas) at the laboratory (1 L) and then pilot (500 L) scales. Unlike most studies that substitute NaHCO_3_ for CO_2_ (25, 26), we used a combined approach to increase CO_2_ capture through two synergistic events: (i) increased microalgal biomass and (ii) CaCO_3_ precipitation through MICP. We designed experiments to measure growth and CaCO_3_ precipitation in IMET1 cultures and revealed that the MadCAP system can capture up to 60% extra CO_2_ capture in the form of aragonite (in addition to algal biomass), with a maximum CO_2_ capture rate of 63.2 g m^−2^ day^−1^. The culture remained stable and robust for over 49 days. We performed 16S rRNA gene sequencing and metagenomic sequencing to characterize bacterial communities and their potential functional contributions to the system. Two novel Patescibacteria were identified as dominant symbionts, and functional analysis revealed genes for plant growth-promotion traits (PGPTs) enriched within this group.

## MATERIALS AND METHODS

### Lab-scale saltwater microalga *N. oceanica* IMET1 culturing and measurements

*N. oceanica* IMET1 was cultured in a modified f/2 medium (27) made with 34 parts per thousand (ppt) artificial seawater. The artificial seawater was obtained from the Aquaculture Research Center at the Institute of Marine and Environmental Technology and is prepared daily from a brine concentrate to mimic the salinity and mineral composition of natural seawater. The artificial seawater was supplemented with 10 mM urea as the nitrogen source. Urea was chosen as it can facilitate MICP and is found to be present in wastewater between 130 and 1,000 mg L^−1^ (28). Under alkaline conditions, the urease enzyme catalyzes the hydrolysis of urea into ammonia (NH_3_) and CO_3_^2-^. The CO_3_^2-^ can be precipitated in the presence of calcium to form CaCO_3_. The seed culture was maintained at room temperature under continuous light illumination of 10–20 μE m^−2^ s^−1^ in Erlenmeyer flasks. Optical density was measured by using a Nanodrop 2000c Spectrophotometer (Thermo Fisher Scientific) at 750 nm (OD_750_). Cell density was measured using a hemocytometer (Hausser Scientific). Dry weight was measured with samples collected on a 45 mm GF/F glass fiber filter (Whatman) as previously described, as they can withstand high temperatures in the drying and combusting procedures (100°C and 500°C, respectively) (24). Key water chemistry indexes (such as pH, salinity, conductivity, calcium, magnesium, and total alkalinity) of algal cultures were performed by ZooQuatic Laboratory, LLC. Langelier Saturation Index (LSI), which indicates the scale-forming potential of water, was calculated based on the following formula (29–31):

LSI=pH−pHs

pHs=(9.3+A+B)−(C+D) where:

$A=(Log_{10}[TDS]-1)/10$

$B=-13.12\times Log_{10}{(}^{\circ }C+273)+34.55$

$C=Log_{10}[Ca^{2+}]-0.4$

$D=Log_{10}[alkalinity]$

TDS (total dissolved solids)=conductivity × 0.67 (the conversion factor for seawater)

In the initial lab-scale testing, different concentrations of NaHCO_3_ (0, 0.02, 0.1, or 0.5 M) were added to the cultures grown in 1 L bubbling columns. The cultures were aerated with 10% CO_2_ that simulated power plant flue gas for 12 days. The aeration was then switched to ambient air for 2 days. In the following optimization test, the cultures containing 0 or 0.02 M NaHCO_3_ were aerated in 1 L bubbling columns under 10% CO_2_ or ambient air. On day 12, the aeration was switched to (or continued using) ambient air for 2 more days.

### Slipstream testing of carbon sequestration by *N. oceanica* IMET1

The slipstream testing was conducted in a 500 L photobioreactor at HY-TEK Bio’s facility at the Baltimore Back River Wastewater Treatment Plant. The 500 L photobioreactor was illuminated by LEDs from the center of the bioreactor and with three sets of external lights (*ca*. 200 µmol m^−2^ s^−1^ light intensity measured at the mid-point of the bioreactor) (Fig. S1 in Supporting Information). The 500 L culture was sparged with simulated boiler flue gas (~5% CO_2_) for 27 days. On day 28, when the algal dry weight plateaued, aeration was switched from 5% CO_2_ to ambient air. On day 31, 0.02 M NaHCO_3_ was added to the culture to assess precipitation. Optical density, pH, dry weight, and ash-free dry weight (AFDW) were measured to monitor growth. The areal productivity was calculated using a bioreactor area ratio of 1:1.2 to represent the bioreactor "facility" square footprint (32).

### Calcium carbonate precipitate analysis

CaCO_3_ precipitate crystallines formed in IMET1 cultures were collected as described previously (24). The collected precipitates were freeze-dried and weighed. The crystalline substance was examined by using a dissection microscope. The composition was determined by X-ray diffraction (XRD) analysis at the X-ray Crystallographic Center at the University of Maryland.

### Prokaryotic community analysis and diversity metrics

DNA extraction, sequencing, and bioinformatic analysis were conducted as previously described using the Quantitative Insights into Microbial Ecology (QIIME2) pipeline (24, 33). For microbiome analysis, the V3-V4 region of the 16S rRNA gene was amplified using the following primers: ‘Illumina 16S V3-V4 primers’ (F: 5'TCGTCGGCAGCGTCAGATGTGTATAA GAGACAGCCTACGGGNGGCWGCAG and R: 5' GTCTCGTGGGCTCGGAGATGTGTAT AAGAGACAGGACTACHVGGGTATCTAATCC); locus-specific sequences are underlined. Based on sequence quality, only the forward reads were analyzed. When analyzing paired reads, only 22% of the reads were retained after filtering compared with 79% when analyzing forward reads alone (Table S1). The quality cutoff was a PHRED score of 24. All sequences identified as algal-derived chloroplast or mitochondrial 16S rRNA gene sequences were removed bioinformatically. The core-diversity-metrics function within Qiime2 was used to analyze alpha-diversity (within each sample) and beta-diversity (between samples). Alpha group significance was calculated using Faith’s Phylogenetic Diversity (34), and beta-group significance was shown using unweighted Unifrac distances (35). Factors such as sampling time, DNA extraction methods, choice of hypervariable region for 16S rRNA gene sequencing, and bioinformatic analysis influence the outcome of the represented taxa (36, 37). All samples were heated at 65°C and underwent bead-beating steps to ensure optimal DNA yields and a higher bacterial diversity (38).

### Metagenomic sequencing and functional gene analysis methods

DNA concentrations were checked by absorbance at 260 nm using a UV–visible spectrophotometer (Nanodrop) and fluorometry (Qubit 1X dsDNA Assay Kit + Qubit fluorometer) and adjusted to the ideal starting quantity of 100–200 fmol (~1,000 ng) in 47 µL nuclease-free water. Sequencing libraries were prepared using the Ligation Sequencing Kit V14 (Oxford Nanopore) following the manufacturer’s protocol with the following adjustments: during the last step of both the End-Prep and Adapter Ligation and Clean-up protocols, the tubes containing AMPure XP beads and the end-prep reaction were incubated at 37°C for 2 min before the eluate was removed. Libraries were made up to 12 µL at 10–20 fmol and loaded onto the GRIDion sequencing platform (Oxford Nanopore) strictly following the manufacturer’s protocol.

For long-read data, quality control analysis before and after filtering, including summary statistics, read length comparison, and average base quality score comparison, was produced using NanoComp (v1.24.0) (39). The long reads were filtered with the chopper v0.8.0 tool of NanoPack2 by applying a minimum Phred average quality score of 10 and a minimum read length of 500 bp. All filtered long reads were mapped against the NCBI nr protein database using Diamond (v2.1.8.162) and MEGAN6 (6UE_6_25_9) using parameters described previously (40). Error-corrected reads and their annotation as GFF3 files were exported from MEGAN6. Bacterial functional counts are normalized to 332,434,816 aligned bases per sample using the MEGAN6 tool compute_comparison with a contamination filter for eukaryotic and viral content. Bar plots displaying function abundances show the top 30 most abundant taxa/functions at a chosen rank or level, per sample or group. If fewer than 30 taxa/functions are present, all were displayed. A diagram of the programs used for analysis is shown in Fig. S2.

### Epifluorescent and scanning electron microscopy

For epifluorescent microscopy, the cells were washed once with 1× phosphate buffer solution (PBS), fixed 1:1 in 4% formaldehyde for 1 h, and washed again. The pellet was resuspended in 1.2 µM 4′,6-diamidino-2-phenylindole (DAPI) and left to soak in the dark for 30 minutes. Cells were filtered onto 0.1 µm black polycarbonate filters (GVS Life Sciences), placed on microscope slides, treated with Diamond Antifade Mountant (Thermo Fisher Scientific), and sealed with a coverslip. Slides were visualized with a Nikon W1 spinning disk microscope at the Confocal Microscopy Facility at the University of Maryland School of Medicine. Microalgal autofluorescence signal was detected at 488 nm (excitation)/ 500–550 nm (emission), and DAPI was detected at 405 nm (excitation)/430–480 nm (emission).

To prepare the samples for scanning electron microscopy (SEM), 2 mL of microalgae-bacteria suspension was centrifuged at 10,000 *g* for 3 min. The pellet was washed once with 1× PBS to ensure the removal of any residual medium. The cells were fixed with 2.5% glutaraldehyde and 4% formaldehyde for 2 h and subsequently washed twice with 1× PBS. Glass coverslips were washed with acetone and coated three times with poly-L-lysine (molecular weight: 70,000–150,000). Coverslips (size: 12 mm, no. 1 thickness) were submerged into the fixed microalgae-bacteria suspension for 30 min at room temperature, followed by an ethanol gradient (30%, 50%, 70%, 80%, 90%, and 100% 3×) for 10 min at each step. Immediately following removal from the 100% ethanol, 20 µL of HMDS (1,1,1,3,3,3-hexamethyldisilazane ≥ 97.0%) was pipetted directly onto the coverslips. Coverslips were mounted onto stubs, sputtered with gold-palladium in a sputter coater (Cressington 108 Manual), and imaged using a FEI Nova NanoSEM 450 at the University of Maryland Baltimore County.

### Digital PCR

Microalgal cultures were normalized to an optical density (absorbance at 750 nm) of 0.8 and processed in two ways. First, 40 mL of the culture was filtered onto a 0.22 µm cellulose acetate filter (Advantec). This size fraction captures both microalgal cells and all bacterial symbionts. Second, additional samples were size fractionated: 30 mL was filtered onto a 0.45 µm cellulose acetate filter (Advantec) and flowthrough was filtered onto a 0.22 µm filter. DNA from all filters was extracted using the PowerWater DNA extraction kit (Qiagen). Digital PCR (dPCR) was used to count the gene copies of the eukaryotic 18S rRNA gene and bacterial 16S rRNA gene, with methods modified from Yu et al. 2022 (41). The QIAcuity EvaGreen PCR Kit (Qiagen) was used to amplify 18S rRNA gene fragments using the eukaryote-specific 528F/706R primer pair: 528 F (5′-GCGGTAATTCCAGCTCCAA-3′); 706 R (5′-AATCCRAGAATTTCACCTCT-3′) (42, 43). The QIAcuity Probe PCR Kit was used to amplify 16S rRNA gene fragments using the bacterial-specific BAC primer/probe set: BAC338F (ACTCC TACGG GAGGC AG; BAC805R (GACTA CCAGG GTATC TAATC C); and Probe- 56FAM 3TAMRA BAC516F (Taqman) (TGCCA GCAGC CGCGG TAATA C) (44). A non-template control (DI water) was included in each run. The reaction and primer/probe mixes were prepared as per the manufacturer’s instructions. The prepared mixtures were loaded onto a QIAcuity 8.5 k 96-well Nanoplate and analyzed in a QIAcuity ONE machine with the following cycling conditions: 95°C for 2 min, 40 cycles of 95°C for 15 s and 60°C for 30 s, and a final step of 35°C for 5 min. To maximize the accuracy of dPCR data output, the samples were not multiplexed, templates were amplified at various 10-fold dilutions, primer and probe concentrations were set as per manufacturer’s instructions, and automatic thresholds as well as Poisson statistics were used during analysis (45).

## RESULTS AND DISCUSSION

### Effects of sodium bicarbonate on the growth of *N. oceanica* strain IMET1

**Fig 1:**
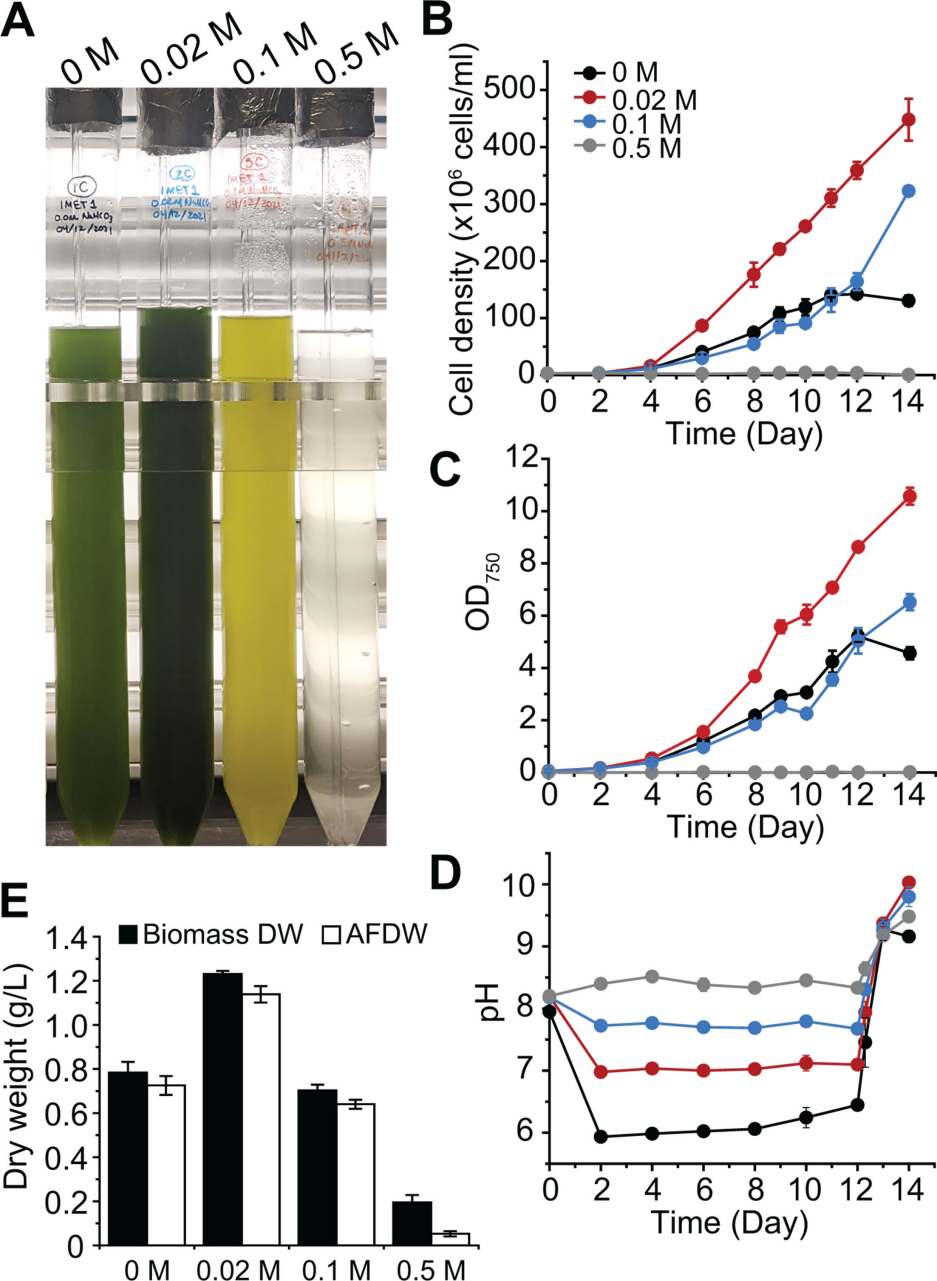
*N. oceanica* IMET1 cultured under 10% CO_2_ with different concentrations of NaHCO_3_. (**A**) *N. oceanica* IMET1 cultures in 1 L column photobioreactors in the presence of 0 M, 0.02 M, 0.1 M, and 0.5 M NaHCO_3_ for 14 days. The growth of *N. oceanica* IMET1 was assessed by cell density (**B**), optical density (**C**), and pH (**D**). At day 12, the CO_2_ supply was shut off, and the cultures were aerated with ambient air for 2 more days. Biomass dry weight and ash-free dry weight (AFDW) were determined on day 12 (**E**). Data represent mean ± standard deviation (SD) from three independent measurements.

In previous work, we tested a range of NaHCO_3_ concentrations on *N. oceanica* growth using ambient air (*ca*. 0.04% CO_2_) (24). By contrast, this study designed all experiments with a primary focus on carbon capture, utilization, and storage using simulated power plant flue gas at 10% CO_2_ and aimed to enhance *N. oceanica* growth and carbon utilization efficiency with flue gas. We first examined the effect of a range of NaHCO_3_ concentrations (0, 0.02, 0.1, and 0.5 M) on the growth of *N. oceanica* IMET1 for 14 days under 10% CO_2_ (Fig. 1). The addition of NaHCO_3_ raises the pH to an alkaline range that is ideal for biomass production, as decreases in pH during CO_2_ bubbling can inhibit microalgal growth (46). Previous studies have focused on growing microalgal cultures with NaHCO_3_ as the sole inorganic carbon source, replacing CO_2_ gas. This study instead aimed to enhance growth and CO_2_ consumption using NaHCO_3_ as supplementation to cultures grown with 10% CO_2_. Our results showed that 0.02 M NaHCO_3_ promoted IMET1 growth and biomass production when grown with 10% CO_2_ (Fig. 1). After day 6, cell density was over 2-fold higher in cultures supplemented with 0.02 M NaHCO_3_ than the cultures with no NaHCO_3_ addition (Fig. 1B). No inhibitory effects were found at 0.1 M NaHCO_3_, whereas at 0.5 M NaHCO_3_, algal growth was inhibited, likely caused by increased osmotic stress (Fig. 1B and C). At day 12, the total biomass dry weight and AFDW of IMET1 cultures given 0.02 M NaHCO_3_ were 1.23 and 1.14 g L^−1^, respectively, whereas at 0 M NaHCO_3_, they were 0.78 and 0.73 g L^−1^, respectively (Fig. 1E). Dry weight in the presence of 0.02 M NaHCO_3_ yielded a 1.6-fold increase over 0 M NaHCO_3_ (Fig. 1E). At day 12, CO_2_ supply was shut off, and the cultures were aerated with ambient air, inducing higher pH and facilitating the precipitation of CaCO_3_. The cultures supplied with 0.02 M and 0.1 M NaHCO_3_ continued to grow exponentially after day 12, likely due to the presence of additional inorganic carbon (HCO_3_^-^) in the medium, whereas the control and 0.5 M NaHCO_3_ culture density leveled off (Fig. 1B and C).

In microalgae, growth responses to NaHCO_3_ seem to be species-specific, and optimal concentrations fall within a range of 0.1–5 g L^−1^ (1.19 mM to 0.060 M) (47). This aligns with the findings of this study, which show 0.02 M being optimal. Bicarbonate-induced growth has previously been observed within the *Nannochloropsis* genus. Under ambient air conditions, *N. salina* cell density was found to increase with additions of 0.5, 1.0, 2.0, and 5.0 g L^−1^ NaHCO_3_ (or 5.59 mM, 11.9 mM, 0.024 M, and 0.060 M NaHCO_3_, respectively) (15). In a separate study (also using ambient air), *N. salina* cultures achieved their highest cell density when supplemented with 1 g L^−1^ NaHCO_3_ (11.9 mM) and showed enhanced total lipid at 2 g L^−1^ NaHCO_3_ (0.024 M) (48). When grown with air sporadically sparged with 5% CO_2_, *N. gaditana* exhibited enhanced growth and lipid content when supplied with 50 mM NaHCO_3_ (16). Our previous work showed that NaHCO_3_ inhibited *N. oceanica* IMET1 growth under ambient air conditions (24), indicating the growth promotion of this strain by NaHCO_3_ only works under high CO_2_ or flue gas conditions.

### Optimizing a saltwater algal carbon sequestration system with NaHCO_3_ and CaCO_3_ precipitation

**Fig 2:**
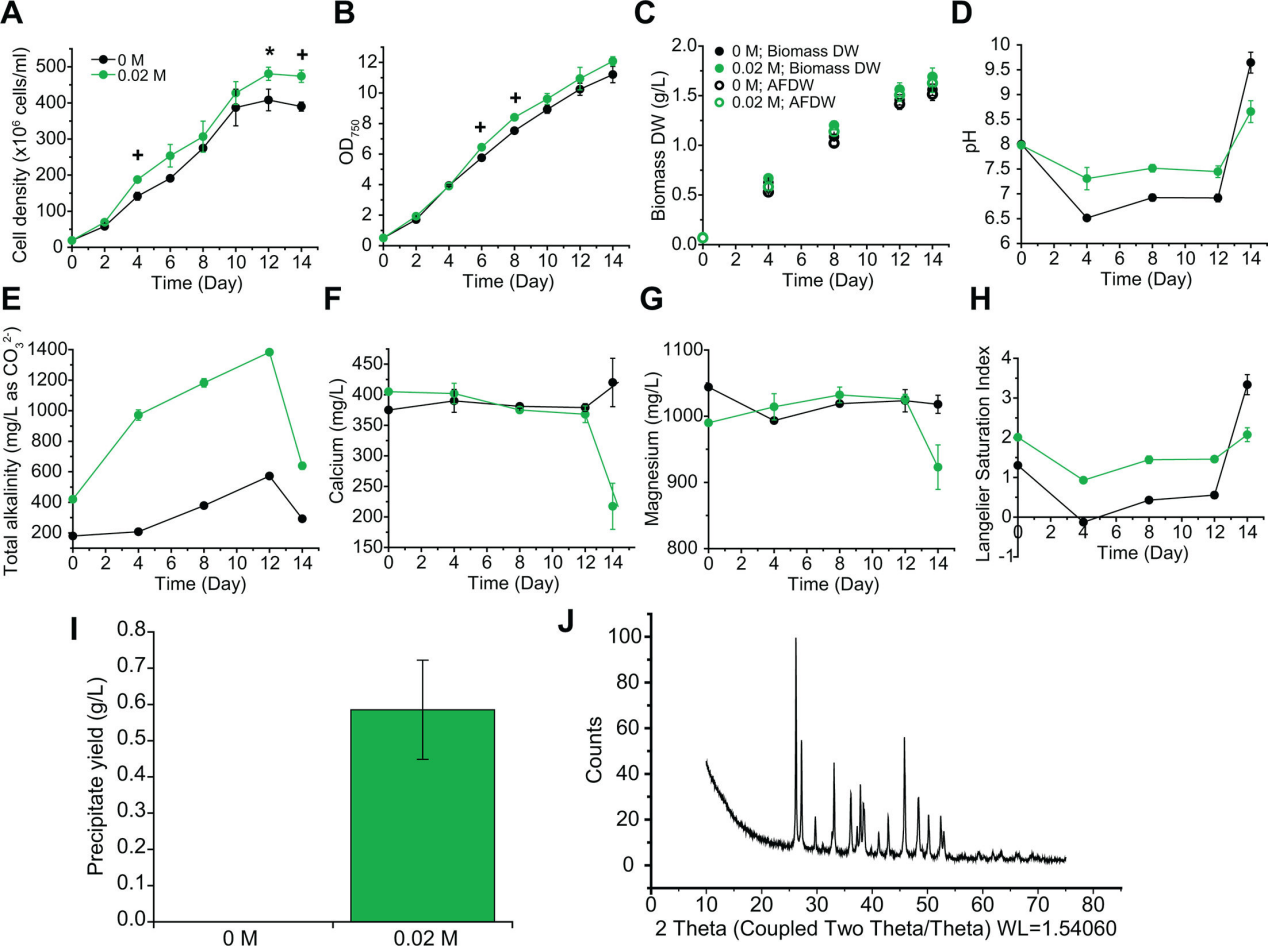
Performance of *N. oceanica* IMET1 cultures under 10% CO_2_ in the presence or absence of 0.02 M NaHCO_3_ in 1 L column photobioreactors. The growth of the cultures was measured by cell density (**A**), optical density (**B**), and biomass dry weight and AFDW (**C**). The water chemistry of pH (**D**), total alkalinity (**E**), calcium concentration (**F**), and magnesium concentration (**G**) in the culture supernatant was measured, and the Langelier Saturation Index (LSI) at 25°C (**H**) was calculated. Optical density was measured with a 10-fold dilution on days–10 and a 100-fold dilution on days 12–14 and calculated by multiplying the readings with the dilution factors. In addition, 10% CO_2_ was switched to ambient air on day 12, and the cultures continued to grow under ambient air until day 14. On day 14, precipitates formed in the cultures were harvested, and the yield of precipitates (**I**) was determined. Data represent mean ± standard deviation (SD) from the three independent measurements. The precipitates were subjected to X-ray diffraction (XRD) analysis (**J**) to determine the composition of the crystalline. The precipitates were composed mostly of CaCO_3_ as aragonites and a small amount of MgCa(CO_3_)_2_.

Next, we further optimized the MadCAP system for maximum carbon capture and precipitate analysis by growing *N. oceanica* IMET1 cultures with 10% CO_2_ and 0.02 M NaHCO_3_, the previously described optimum concentration. We also measured the changes in medium alkalinity and chemistry to determine how these conditions affect the precipitation of CaCO_3_. It has been shown that an increase in the initial cell density can lead to higher final cell density and higher biomass productivity due to reduced photoinhibition and faster growth rates at the early growth stage (Days 0 –4) (49). In this optimization effort, the initial cell density was increased by 6-fold (initial cell density as 1.9 × 10^7^ cells/mL vs. 3.1 × 10^6^ cells/mL) compared with the initial test, which resulted in increased final cell density and biomass dry weight yield. As expected, algal cell density was significantly higher when cultures were given 0.02 M NaHCO_3_ when compared with the control (0 M) (Fig. 2A). On day 4, cell density of the 0.02 M cultures was 1.3-fold higher than the control, and at day 12, the cell density of the 0.02 M cultures was 1.2-fold higher than the control (Fig. 2A). However, biomass dry weight production in the presence and the absence of NaHCO_3_ was similar throughout the experiment (Fig. 2C). Total dry weight and AFDW at the end of the growth phase under 10% CO_2_ (day 12) for the 0.02 M cultures were 1.54 and 1.47 g L^−1^, respectively, whereas the control culture yielded 1.49 and 1.41 g L^−1^, respectively (Fig. 2C). After cultures were switched from 10% CO_2_ to ambient air, AFDW further increased to 1.52 g L^−1^ and 1.47 g L^−1^ (0.02 M NaHCO_3_ and control, respectively) (Fig. 2C). Compared with the initial test (Fig. 1), the growth rate and biomass yield were higher in the optimized system (Fig. 2). The highest dry weight achieved was 1.52 g L^−1^ (AFDW) with 10% CO_2_ and 0.02 M NaHCO_3_ supplementation. Because we grew non-axenic algal cultures, we observed some differences in cell growth and dry weight between cultures in Fig. 1 and 2, whereas the observation of growth promotion by NaHCO_3_ stayed the same.

After CO_2_ shut-off, the culture pH increased quickly from 7.3 to over 8.6 on day 14 (Fig. 2D), likely because of the depletion of dissolved CO_2_ and H^+^ through reaction (i): ${CO_{2}{+H}_{2}O⇌H}_{2}{CO}_{3}⇌H^{+}+{HCO}_{3}^{-}$. We measured the total alkalinity and the concentration of calcium (Ca^2+^) and magnesium (Mg^2+^) in the cultures to calculate the Langelier Saturation Index (LSI) to determine if water is corrosive (LSI < 0) or scale-forming (LSI > 1). LSI quickly increased following CO_2_ shut-off (Fig. 2H). After CO2 shut-off, the level of total alkalinity (as CO_3_^2-^) as well as the level of Ca^2+^ and Mg^2+^ decreased (Fig. 2E through G). Simultaneously, precipitation of CaCO_3_ and MgCO_3_ occurred as a consequence. As a result, 0.59 ± 0.14 g L^−1^ precipitates were recovered in the cultures with 0.02 M NaHCO_3_ (Fig. 2I). The collected precipitates were subjected to X-ray diffraction analysis (XRD), and the crystalline material was found to contain >90% aragonite (CaCO_3_) and <10% magnesium calcite (MgCa(CO_3_)_2_) (Fig. 2J; Fig. S4). To calculate the equivalent CO_2_ consumption rate, the assumptions are: 183 g of CO_2_ will result in 100 g algae or 416 g CaCO_3_; to produce 100 g CaCO_3_ (equivalent to 1 mole CaCO_3_), 44 g CO_2_ (equivalent to 1 mole CO_2_) is needed. The formation of CaCO_3_ precipitates in the cultures with 0.02 M NaHCO_3_ resulted in 9.3% extra CO_2_ consumption. Algal cell surfaces and the extracellular polymeric substances in the microalgal biofilm provide substrates that facilitate the nucleation and precipitation of CaCO_3_ (50). The formation of CaCO_3_ in microalgal cultures, mediated by MICP, has been studied in several algal species, including *N. oceanica* IMET1 (18, 19, 24). Although many studies have investigated MICP within microalgae that form carbonate skeletons, coccolithophores (51, 52), few have paired CaCO_3_ formation with a lipid-rich alga that has the ability to grow under high CO_2_ conditions. Our focus on MICP is 2-fold: first, carbonate formation enables more atmospheric carbon to be captured in a stable, recalcitrant form (9.3% extra CO_2_ consumption). Second, CaCO_3_ is a valuable precursor for various industries (53).

We also tested the carbon sequestration of IMET1 using ambient air (Text S1). For consistency, the cultures were again set up in the presence or the absence of 0.02 M NaHCO_3_ and grown for 14 days. By day 4, the growth of IMET1 cultures with air was greatly promoted by 0.02 M NaHCO_3_ (Fig. S3). However, cell density and biomass concentrations of cultures grown with air were much lower compared with that of 10% CO_2_.

### Up to 60% extra CO_2_ was captured by marine microalgae in a scaled-up (500 L) system

**Fig 3:**
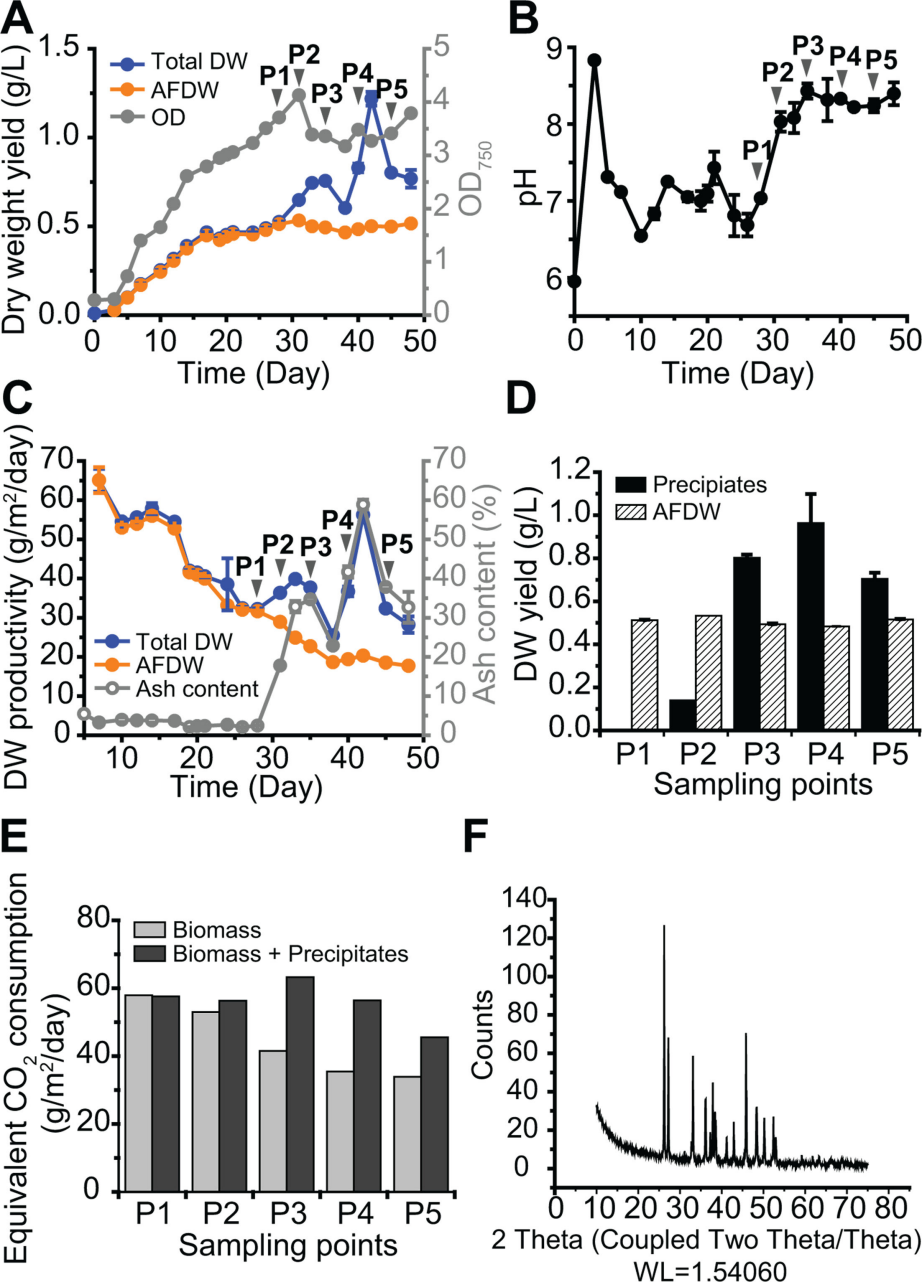
Performance of *N. oceanica* IMET1 cultures under 5% CO_2_ in a 500 L photobioreactor. The growth (**A**) and pH (**B**) of the cultures were monitored. The growth measurements included the average productivity of dry weight (DW) and ash-free dry weight (AFDW) (**C**), the yield of precipitates and AFDW (**D**), and the equivalent CO_2_ consumption rate of algal biomass production and the combined production of biomass plus the CaCO_3_ precipitates (**E**) of sampling points P1–P5 were calculated. The precipitates were subjected to X-ray diffraction (XRD) analysis (**F**) to determine the composition of the crystalline CaCO_3_ as aragonite. Data represent mean ± standard deviation (SD) from the three independent measurements. On day 28, the sparging of 5% CO_2_ was switched to ambient air, and 0.02 M NaHCO_3_ was added to the culture on day 31. After the sparging switching to ambient air, precipitates in the culture were collected at five time points (downward triangles in A, **B, and C**): P1 (Day 28, the sparging switched from CO_2_ to ambient air), P2 (Day 31, addition of 0.02 M NaHCO_3_ to the culture), P3 (Day 35, 4 days after addition of NaHCO_3_), P4 (Day 40, 9 days after addition of NaHCO_3_), and P5 (Day 45, 14 days after the addition of NaHCO_3_).

In the optimized set-up (Fig. 2), the improvement of *N. oceanica* IMET1 growth was marginal, but the precipitate yield was greatly enhanced with the addition of NaHCO_3_. We aimed to separate the effect of (1) pH increase by switching the CO_2_ supply to ambient air, and (2) NaHCO_3_ addition on extra CO_2_ capture. Thus, we applied these two changes on separate days when microalgal growth started to plateau. A culture of IMET1 was set up in HY-TEK Bio’s 500 L bioreactor (Fig. S1). During the first 15 days of growth, biomass dry weight and AFDW rapidly increased (up to 0.4 g L^−1^ AFDW) (Fig. 3A), resulting in a greater than 50 g m^−2^ day^−1^ average AFDW productivity (Fig. 3C). After day 15, IMET1 growth rate declined, likely due to nutrient limitation. To test this, 0.9 g L^−1^ NaNO_3_ was added, and the culture growth resumed, albeit at a slower rate. On day 28, the average AFDW productivity was 31.5 g m^−2^ day^−1^ (Fig. 3C). On day 28, the CO_2_ supply was shut off, and the culture was grown with ambient air (Point 1 for precipitate analysis, P1). On day 31 (Point 2 for precipitate analysis, P2), 0.02 M NaHCO_3_ was added, and the culture continued for 4 more days before the third collection for precipitate analysis (P3). Two additional precipitate collection points were on day 40 (P4) and day 45 (P5). After CO_2_ shut off, the culture pH increased from about 7–8 (Fig. 3B), whereas after adding NaHCO_3_, the culture pH increased to 8.2. At the same time, precipitate yield greatly increased, from 26.2% (of algae ADFW) at P2 (day 31) to 160.4% at P3 (day 35) (Fig. 3D). The increase in precipitation corresponded to the increase of ash content in the culture (Fig. 3C). Adding 0.02 M NaHCO_3_ at day 31 did not improve the growth as the optical density had already plateaued, but the addition of NaHCO_3_ assisted CaCO_3_ precipitation. The precipitate yield remained the same as P3 at P4 and P5 (Fig. 3D). We calculated the areal CO_2_ capture rate (g m^−2^ day^−1^) by converting CO_2_ captured as CaCO_3_ into CO_2_ capture rate. From P2 (Day 31) to P3 (Day 35), the extra CO_2_ captured as CaCO_3_ greatly increased from 6% to 52%. There was up to 60% extra CO_2_ capture (on Day 40) with a maximum CO_2_ capture rate of 63.2 g m^−2^ day^−1^ (on Day 35) (Fig. 3E). XRD analysis confirmed the precipitates were aragonite (Fig. 3F).

In corroboration with this study, our previous work showed CO_2_ fixation efficiency increased with CaCO_3_ precipitation, and calcite was recovered from IMET1 grown with air in a 340 L photobioreactor (24). Yu et al. 2022 also found calcite formation on the cell surface of *Chlorella* sp. HS2 when cultures were supplemented with CaCl_2_ in a 20 L photobioreactor (54). CaCl_2_ supplementation leading to CaCO_3_ formation also increased biomass and lipid production in the aforementioned *Chlorella* sp. and in *Nannochloropsis oleoabundans*, which could be attributed to increased light availability driven by the multiple light scattering processes of CaCO_3_ crystal formation (55). We did not need to add calcium into our system as CaCl_2_ is present in the artificial seawater used to make the algal growth medium.

### Bacteria associated with *N. oceanica* IMET1 and the algal carbon sequestration system

Symbiotic associations between microalgae and bacteria have become a focus in microalgal biotechnology due to the various advantages offered to both organisms (56). In this study, algal-bacterial associations have been methodologically separated into two categories: “closely associated” symbionts, including attached symbionts and endosymbionts, were caught on a 0.45 µm filter along with microalgal cells. Subsequently, “free-living” symbionts pass through the 0.45 µm filter and are collected on a 0.22 µm filter. Other studies have fractionated bacteria using similar methods, albeit with various filter sizes, for example, 5 µm (57) and 3 µm (58). Considering that *N. oceanica* IMET1 can be as small as 2.6 µm in size (Fig. S5), 0.45 µm was deemed an appropriate fractionation between algae and free-living bacteria.

**Fig 4:**
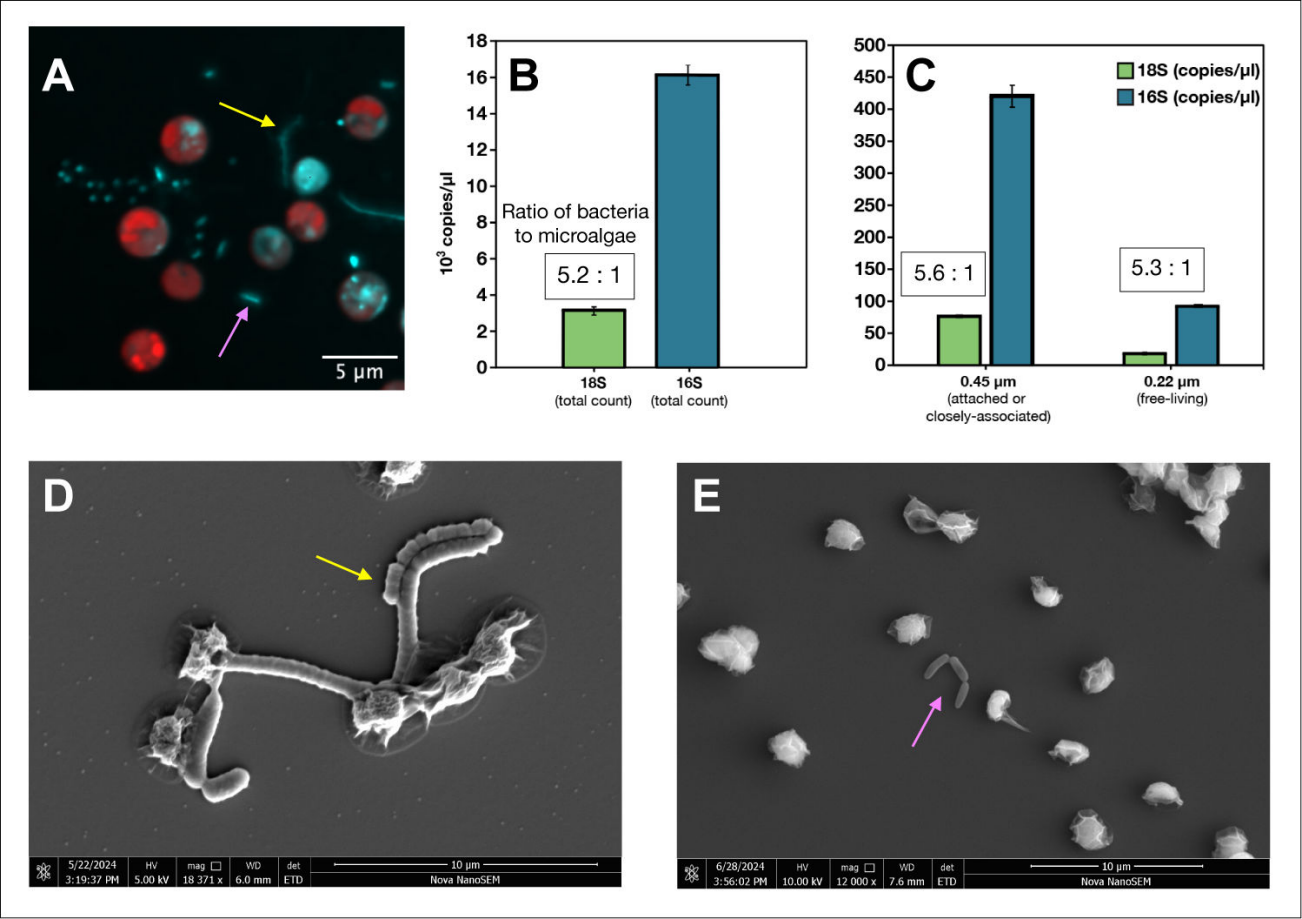
*N. oceanica* strain IMET1 and its bacterial symbionts. (**A**) Epifluorescent microscopy showing microalgal autofluorescence in red and bacteria stained with DAPI in blue. DAPI also stains nucleic acid inside of the microalgal cells, and thus, the fluorescence signal from inside an algal cell is not considered to be a bacterial endosymbiont. Yellow arrows indicate long chains of closely associated bacteria, and pink arrows indicate free-living bacteria. (**B**) Digital PCR data displaying counts of microalgal 18S rRNA genes (green) and bacterial 16S rRNA genes (blue) with the bacteria-to-microalgae ratio being 5:1. (**C**) Digital PCR data of 18S and 16S rRNA genes after cultures were filtered onto 0.45 µm filters with flow-through and then filtered on 0.22 µm; ratio remains 5:1. (**D**) Scanning electron micrograph (SEM) of closely associated bacteria with *N. oceanica* and (**E**) SEM of free-living bacteria with *N. oceanica*.

Microalgae and bacteria were imaged using epifluorescent microscopy and SEM (Fig. 4). We imaged both attached symbionts (yellow arrows, Fig. 4D) and free-living symbionts (pink arrows, Fig. 4E). Ratio of bacterial cells to microalgal cells was found to be 5:1 as determined by dPCR (Fig. 4B and C). This ratio held consistent regardless of whether the microalgal cultures were filtered onto a 0.45 µm filter, containing bacteria considered closely associated symbionts (Fig. 4B) or onto a 0.22 µm filter, containing free-living symbionts (Fig. 4C). There were fewer bacteria found at the 0.22 µm fraction (925,000 16S copies/µL) than at the 0.45 µm (420,000 16S copies/µL) (Fig. 4C). This indicates that *N. oceanica* IMET1 likely has more closely associated symbionts than free-living symbionts. The decrease could also potentially come from some free-living bacteria becoming stuck on the 0.45 µm filters, although precautions were taken to stop filtering as soon as the 0.45 µm pores became saturated with microalgae.

**Fig 5:**
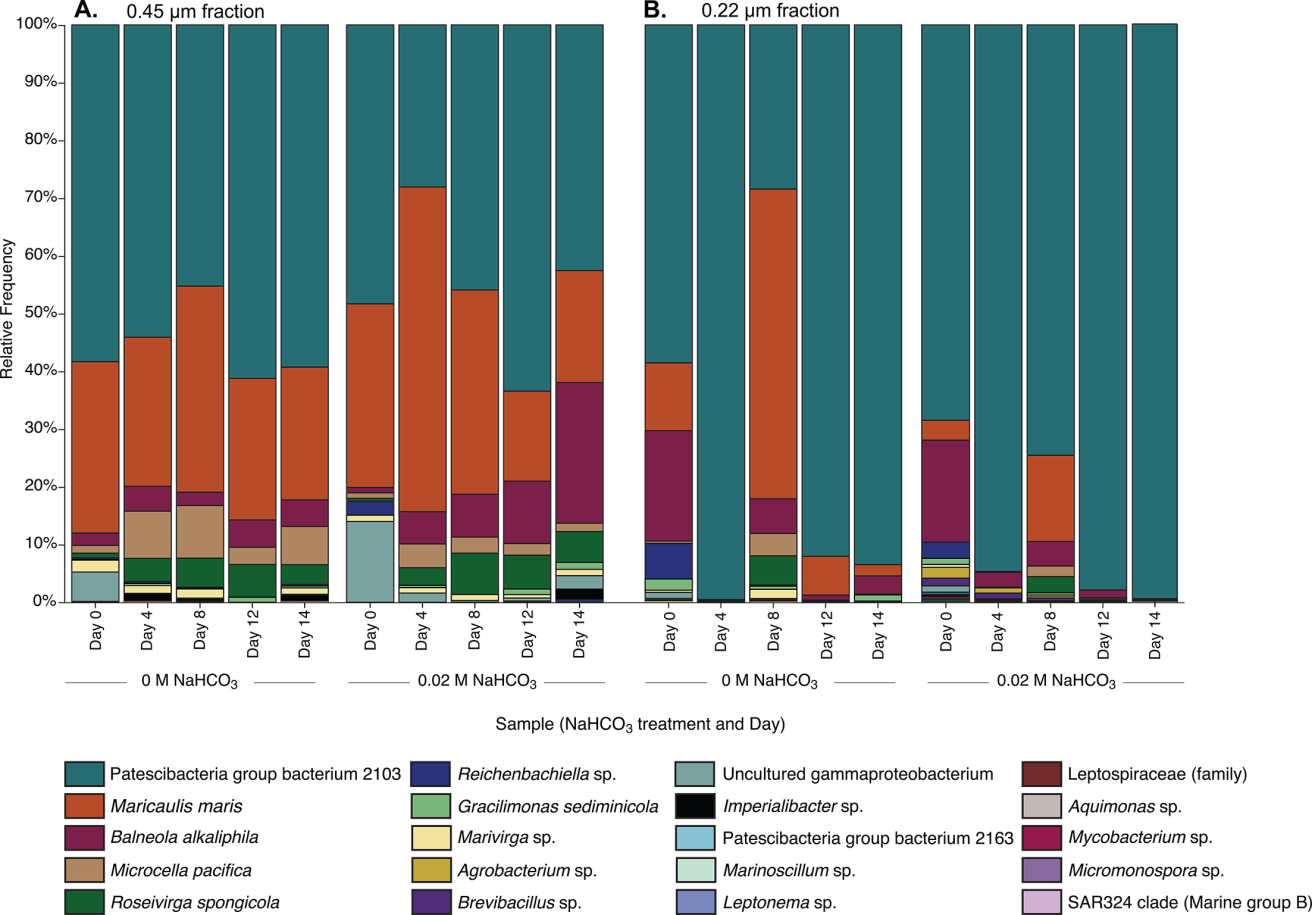
Relative abundance plots of the closely associated prokaryotic community (0.45 µm) and free-living prokaryotic community (0.22 µm) of *N. oceanica* IMET1 through 16S rRNA gene sequence analysis. IMET1 was grown at 1 L scale with 10% CO_2_ for 12 days, then grown with ambient air until day 14. Cultures were supplemented with either no addition (0 M) or 0.02 M addition of NaHCO_3_. 16S rRNA gene sequences from *N. oceanica* chloroplasts were removed bioinformatically.

Figure 5 shows the bacterial communities (16S rRNA barcode analysis) recovered from *N. oceanica* cultures bubbled with 10% CO_2_ (the growth experiment shown in Fig. 2). Table 1 lists the values for percent relative abundance of phyla recovered. Alpha and beta diversity metrics are described in Text S2 and Fig. S7 to S13. Our previous bacterial community analysis of the cultures grown with ambient air revealed the dominance of an uncultured bacterium clone related to the novel and enigmatic phylum, Patescibacteria (24). Interestingly, this bacterium was again found in *N. oceanica* cultures grown with 10% CO_2_ (Fig. 5) and makes up approximately 80% of the bacterial community (Table 1). Metagenomic sequencing revealed that this dominant taxon was indeed two bacteria (Jonas et al. submitted for publication), one of them dominant across all samples, regardless of NaHCO_3_ input, day of experiment, or filter fraction size (Fig. 5- dark teal bars). Other bacterial species included *Balneola alkaliphila, Microcella pacifica*, and *Gracilimonas sediminicola*, all of which belong to genera that have been previously found within a consortium of microorganisms capable of precipitating CaCO_3_ (59). *Gracilimonas* spp. (of the order Balneolales) have been recovered from various hypersaline soda lakes and CaCO_3_ formations (60–62). Other notable symbionts of IMET1 were *Roseivirgia spongicola*, *Marivirgia* sp., an uncultured Gammaproteobacterium, and *Maricaulis maris*, a biofilm-forming bacterium found in oligotrophic waters (63). Finally, *Leptonema* sp. sequence was repeatedly detected in association with *N. oceanica* and was imaged by SEM (Fig. S6). When interpreting these results, we note that abundances are relative, that certain taxa may have primer and amplification biases, and that the observed communities are a snapshot of the true underlying abundances throughout the experiment. The bacterial community associated with IMET1 has been analyzed once before using denaturing gradient gel electrophoresis (DGGE) and 16S rRNA gene-based clone library construction (64). Through both methodologies, Alphaproteobacteria (48%) and Bacteroidetes (28%) dominated the culturable bacterial community. Although Alphaproteobacteria and Bacteroidetes were abundant in our data set (9.48% and 8.31%, respectively), Patescibacteria dominated at 79.7% (Table 1). Shi et al. (65), pursuing wastewater treatment using microalgal-bacterial granular sludge, found that the process was enhanced with the addition of NaHCO_3_ and created a more favorable environment for Proteobacteria, Chloroflexi, and Cyanobacteria (65). We found neither Chloroflexi nor Cyanobacteria associated with *N. oceanica* IMET1.

**TABLE 1:** Percent relative abundance at the phylum level of bacteria associated with *N. oceanica* IMET1 bubbled with 10% CO_2_^*a*^

Phylum	Relative abundance (%)
Patescibacteria	79.70
Proteobacteria [Alphaproteobacteria] [Gammaproteobacteria]	9.88 [9.48] [0.40]
Bacteroidota	8.31
Actinobacteriota	1.63
Firmicutes	0.34
Spirochaetota	0.12
SAR324 clade	0.02

^*a*^ Values reflect average read counts across all samples shown in Fig. 5.

### Functional gene analysis suggests that bacteria encode for functions that could enhance microalgal growth

**Fig 6:**
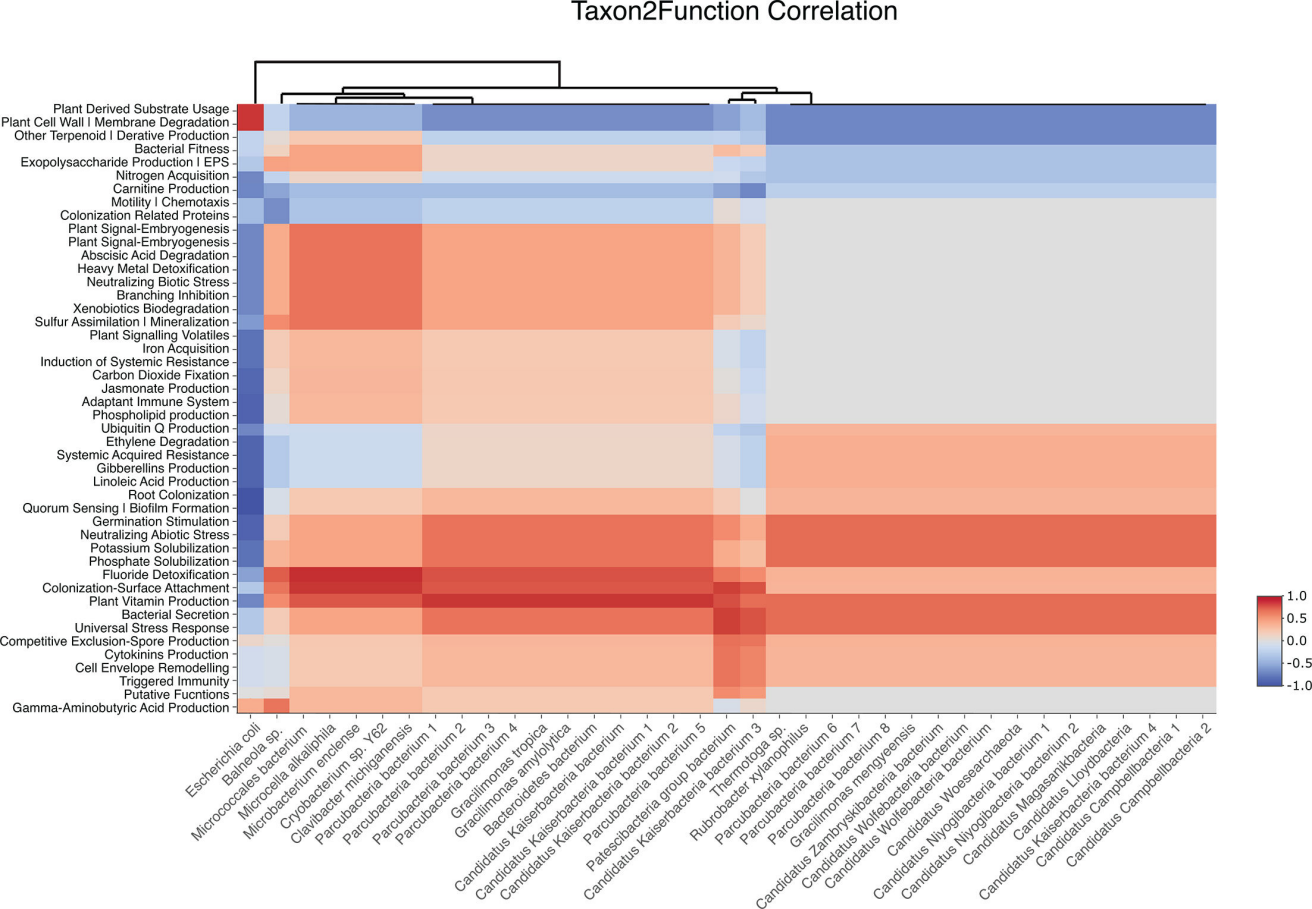
Heatmap displaying the correlation values of the most abundant species with the top functions within PGPT Level 3 using Spearman’s rank correlation coefficient. A table displaying the NCBI’s current full name of organisms abbreviated in heatmap (e.g., Parcubacteria bacterium1) is shown in Fig. S13D.

The PLant-associated BActeria web resource (PLaBAse) (66) assigns a functional annotation of plant growth-promotion traits (PGPTs), which are insightful for understanding the role of bacteria on carbon capture through microalgal growth enhancement. Annotation of PGPTs as well as KEGG (Kyoto Encyclopedia of Genes and Genomes [67]) functional annotations are described in Text S3 and Fig. S14. Several PGPT level 3 functions were found to be highly correlated (>0.5 correlation value) with various taxa found within our system (Fig. 6). Fluoride detoxification, colonization-surface attachment, and plant vitamin production genes were positively correlated with the presence of several bacteria including *Microcella pacifica*, *Gracilimonas* sp., and Patescibacteria group bacteria, suggesting that they may promote algal growth through secretion of vitamins (Fig. 6). The presence of fluoride detoxification genes is in line with the fact that fluoridated tap water is used to prepare algae medium. Furthermore, algae, including *N. oceanica*, are known to release highly labile organic carbon that can then be consumed by attached or nearby bacteria (68, 69). In return, bacteria can provide algae with vitamins, such as B_12_, and can promote microalgal growth (70, 71). There were no relevant findings when looking at bacterial genes related to carbon fixation, although we hypothesize that bacterial symbionts may contribute to microalgal growth enhancement through mechanisms such as vitamin production and secretion (Fig. 6). There are two possibilities as to how bacteria contribute to carbon capture and/or MICP, the first being that the bacteria help microalgal growth as seen across multiple studies (72). The second possibility is that the bacteria themselves are contributing to CaCO_3_ precipitation. The literature indicates *Gracilimonas* sp. and *Microcella* sp. are often found in carbonate formations (59), indicating that they could be key players within our system, but we cannot claim this is occurring in our system as of yet.

The bacteria that seem to potentially contribute the most functionality in this study are members of the Patescibacteria group. These are also the most abundant taxa found in the 16S sequencing data (Fig. 5). Many studies have shown that Patescibacteria often attach to the surface of other bacteria (hence the high correlation with colonization-surface attachment genes, Fig. 6) (73, 74). It must be noted that any taxa labeled as “Candidatus Kaiserbacteria” and “Parcubacteria” could be the same organisms as “Patescibacteria group bacterium” due to the current phylogenetic difficulty of classifying members of Patescibacteria. The phylum is poorly classified and largely uncultured, and the extent of its subclassified diversity remains unresolved (75, 76). The dominance of Patescibacteria in our microalgal cultures warrants their further investigation as key symbionts that may impact algal growth and/or carbon sequestration efficiency.

## Data Availability

The BioProject number for 16S rRNA gene sequencing data is PRJNA1234205. The Sequence Read Archive (SRA) number for metagenomic sequencing is SRR31285674, and the MAG accession numbers are CP174359 and CP174360.

## SUPPLEMENTAL MATERIAL

Supplemental material aem.00133-25-s0001.docx Figures S1 to S14, Table S1, and Text S1 to S3.

ASM does not own the copyrights to Supplemental Material that may be linked to, or accessed through, an article. The authors have granted ASM a non-exclusive, world-wide license to publish the Supplemental Material files. Please contact the corresponding author directly for reuse.
