# Supplementary material for: Nannochloropsis oceanica IMET1 and its bacterial symbionts for carbon capture, utilization, and storage: biomass and calcium carbonate production under high pH and high alkalinity
Source: Appl Environ Microbiol. 2025 Apr 17;91(5):e00133-25. doi: 10.1128/aem.00133-25 (PMC12093951; doi:10.1128/aem.00133-25)
Supplement: Supplemental material — Figures S1 to S14, Table S1, and Text S1 to S3. [file aem.00133-25-s0001.docx]

**Supporting Information for**

***Nannochloropsis oceanica* IMET1 and its bacterial symbionts for carbon capture, utilization, and storage: Biomass and calcium carbonate production under high pH and high alkalinity**

Lauren Jonas^1‡^, Yi-Ying Lee^1‡^, Robert Mroz^2^, Russell T. Hill^1*^ and Yantao Li^1*^

^1^Institute of Marine and Environmental Technology, University of Maryland Center for Environmental Science, Baltimore Maryland, USA

^2^HY-TEK Bio, LLC, Baltimore, Maryland, USA.

^*^Corresponding Author

^‡^ These authors contributed equally


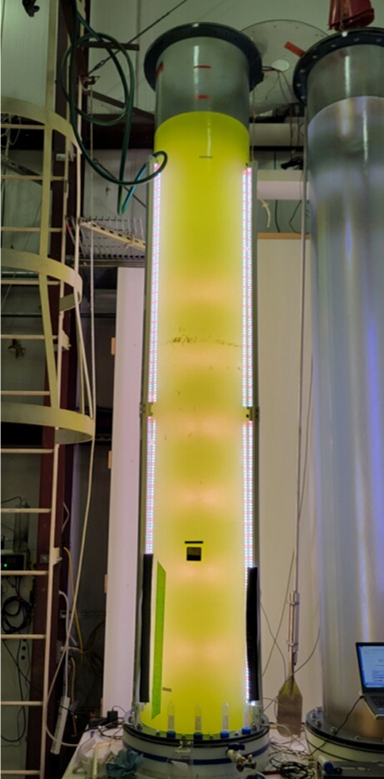


**Figure S1**. Photo of 500 L photobioreactor filled with *Nannochloropsis oceanica* strain IMET1 at HyTek Bio in Baltimore, Maryland.

**Table S1**. Denoising statistics table after performing read joining and quality filtering. Table shows total number and percentage of sequences that were retained with each method.

|  | Total number of sequences to be analyzed after filtering | | Percentage of sequences analyzed from original total | |
| --- | --- | --- | --- | --- |
| Sample | paired | forward  only | paired | forward only |
| 0.22_0.02M_Day0 | 11,875 | 44,963 | 20.44 % | 77.38 % |
| 0.22_0.02M_Day12 | 10,482 | 47,129 | 17.64 % | 79.31 % |
| 0.22_0.02M_Day14 | 0 | 0 | 0 % | 0 % |
| 0.22_0.02M_Day4 | 16,883 | 58,902 | 23.47 % | 81.88 % |
| 0.22_0.02M_Day8 | 21,797 | 62,341 | 30.14 % | 86.21 % |
| 0.22_0M_Day0 | 19,238 | 50,738 | 30.52 % | 80.49 % |
| 0.22_0M_Day12 | 12,604 | 46,815 | 21.66 % | 80.47 % |
| 0.22_0M_Day14 | 10,048 | 36,909 | 22.14 % | 81.34 % |
| 0.22_0M_Day4 | 8,039 | 35,110 | 18.38 % | 80.26 % |
| 0.22_0M_Day8 | 29,541 | 91,365 | 26.93 % | 83.28 % |
| 0.45_0.02M_Day0 | 21,741 | 64,171 | 28.86 % | 85.17 % |
| 0.45_0.02M_Day12 | 11,416 | 63,287 | 15.74 % | 87.25 % |
| 0.45_0.02M_Day14 | 16,175 | 82,142 | 17.32 % | 87.94 % |
| 0.45_0.02M_Day4 | 26,832 | 78,576 | 29.47 % | 86.29 % |
| 0.45_0.02M_Day8 | 20,786 | 70,259 | 25.34 % | 85.66 % |
| 0.45_0M_Day0 | 33,641 | 163,029 | 17.85 % | 86.5 % |
| 0.45_0M_Day12 | 11,170 | 38,453 | 23.94 % | 82.41 % |
| 0.45_0M_Day14 | 31,558 | 102,668 | 26.16 % | 85.1 % |
| 0.45_0M_Day4 | 37,455 | 126,101 | 25.56 % | 86.07 % |
| 0.45_0M_Day8 | 39,765 | 129,695 | 26.06 % | 84.98 % |
| **Average** | **19,552** | **69,632** | **22.38%** | **79.40%** |

**Figure S2.** Pipeline depicting filtering methods, sequencing information, and bioinformatic programs used for 16S rRNA gene analysis and metagenomic functional gene analysis.

**Text S1. *N. oceanica* grown with ambient air and no (0 M) or 0.02 M NaHCO_3_**

In contrast to the cultures grown with 10% CO_2_, the growth of IMET1 cultures with air was greatly promoted by 0.02 M NaHCO_3_ (Figure S3). The difference between cell density of the cultures with and without NaHCO_3_ significantly increased after Day 4 (Figure S3A). The cell density of the cultures containing NaHCO_3_ was 1.86-fold higher than the control cultures on Day 10 when the cultures containing NaHCO_3_ started to plateau (Figure S3A). Similarly, the biomass dry weight production was significantly higher at all time points after Day 4. At the end of the experiment (Day 14), the total dry weight and the AFDW of the culture containing NaHCO_3_ were 1.63-fold and 1.50-fold higher than the control culture, respectively (Figure S3C). Although NaHCO_3_ substantially promoted IMET1 growth under ambient air conditions, the cell density and biomass concentrations of IMET1 cultures under 10% CO_2_ was considerably better. For example, at Day 14, cell counts were 4.74 x 10^8^ cells ml^-1^ (10% CO_2_) vs. 8.32 x 10^7^ cells ml^-1^ (Air) and AFDW was 1.52 g L^-1^ (10% CO_2_) vs. 0.30 g L^-1^ (Air).

The pH of the cultures in the presence or absence of NaHCO_3_ increased rapidly and remained above 9.5 after Day 4 (Figure S3D), which led to high LSI values (>3) (Figure S3H). As the growth progressed, the level of calcium in the cultures at 0.02 M NaHCO_3_ gradually decreased and at Day 14 was half of the initial level (Figure S3F). Total alkalinity behaved similarly (Figure S3E). As a result, 0.67 ± 0.05 g L^-1^ precipitate was recovered in the cultures in the presence of NaHCO_3_ (Figure S3I). XRD analysis revelated precipitates were CaCO_3_ in the form of monohydrocalcite, Ca(CO_3_)H_2_O (Figure S3J).


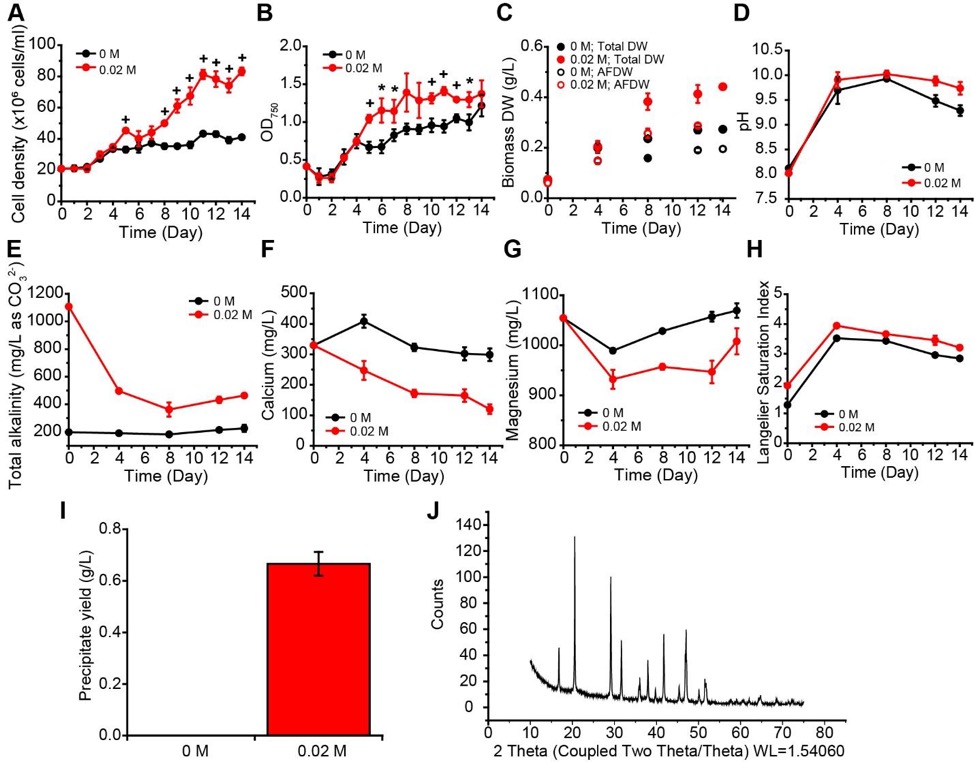


**Figure S3.** Performance of *Nannochloropsis oceanica* IMET1 cultures under ambient air in the presence or absence of 0.02 M NaHCO_3_ in 1 L column photobioreactors. The growth of the cultures was measured by cell density (A), optical density (B), biomass dry weight and ash-free dry weight (AFDW) (C). During the growth course, the water chemistry of pH (D), total alkalinity (E), calcium concentration (F), magnesium concentration (G) in the culture supernatant were measured, and the Langelier Saturation Index (LSI) at 25^o^C (H) was calculated. Precipitates formed in the cultures were harvested at Day 14, and the yield of precipitates (I) was determined. Data represents mean ± standard deviation (SD) from the three independent measurements. The precipitates were subjected to X-ray diffraction (XRD) analysis (J) to determine the composition of the crystalline as CaCO_3_ as monohydrocalcite.


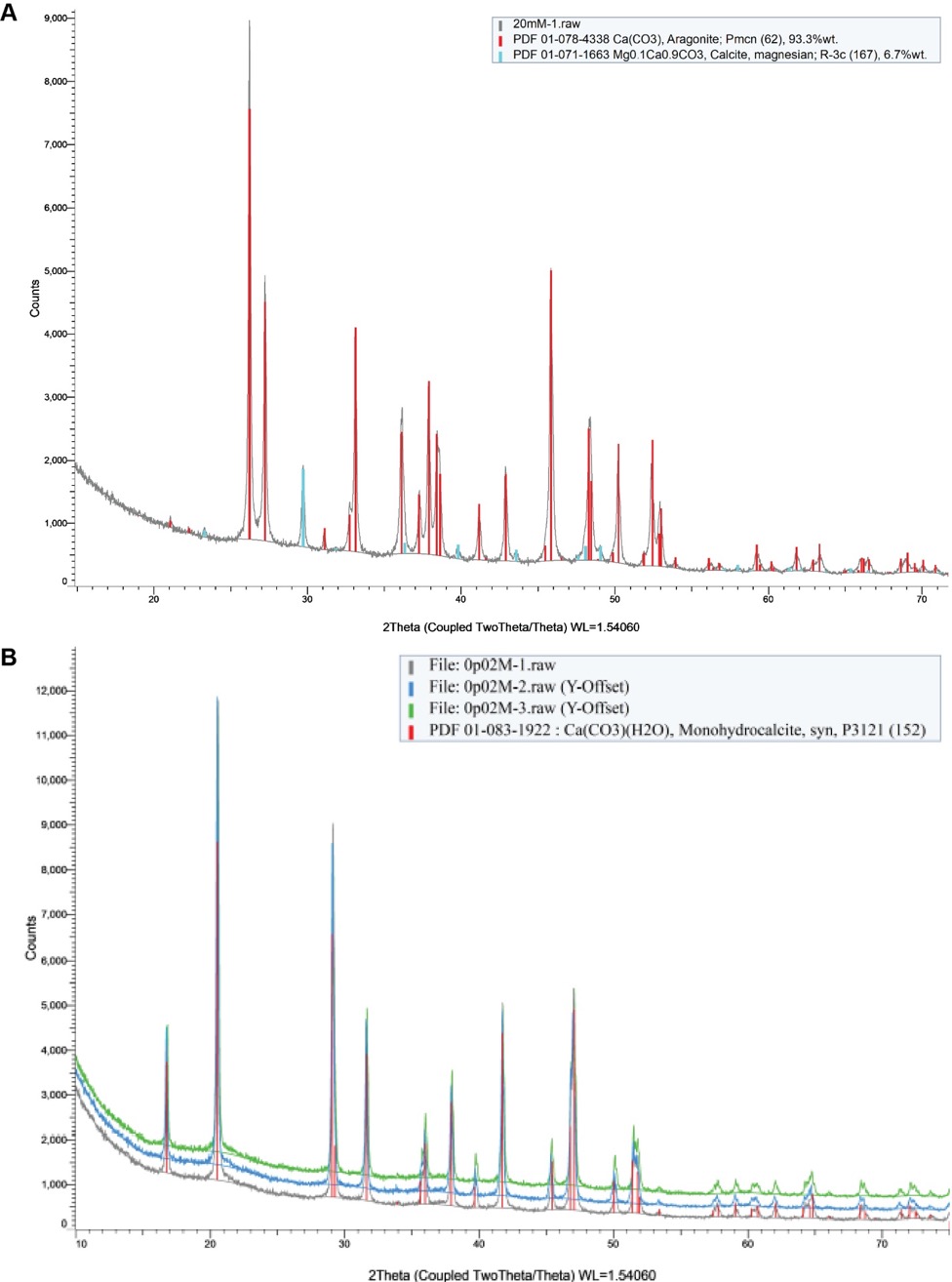


**Figure S4.** XRD spectra overlays between the sample precipitates and the crystalline references. (A) XRD spectra of the precipitates collected from the cultures under 10% CO_2_ (gray), aragonite (red) and magnesium calcite (blue). (B) XRD spectra of the precipitates collected from the cultures under ambient air (gray, blue and green) and monohydrocalcite (red).


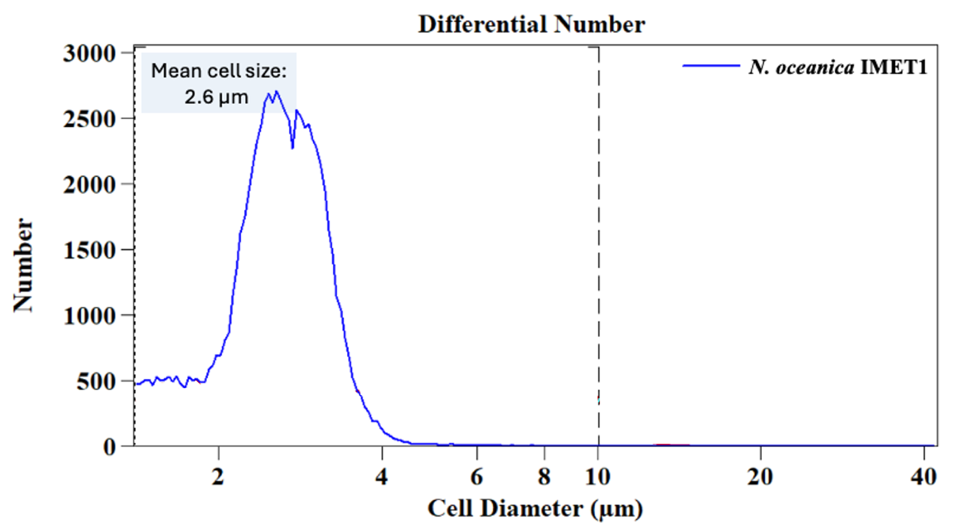


**Figure S5.** Size distribution of *N. oceanica* IMET1 using a Coulter Counter. Peaks indicate that IMET cells have a mean diameter of 2.6 µm.


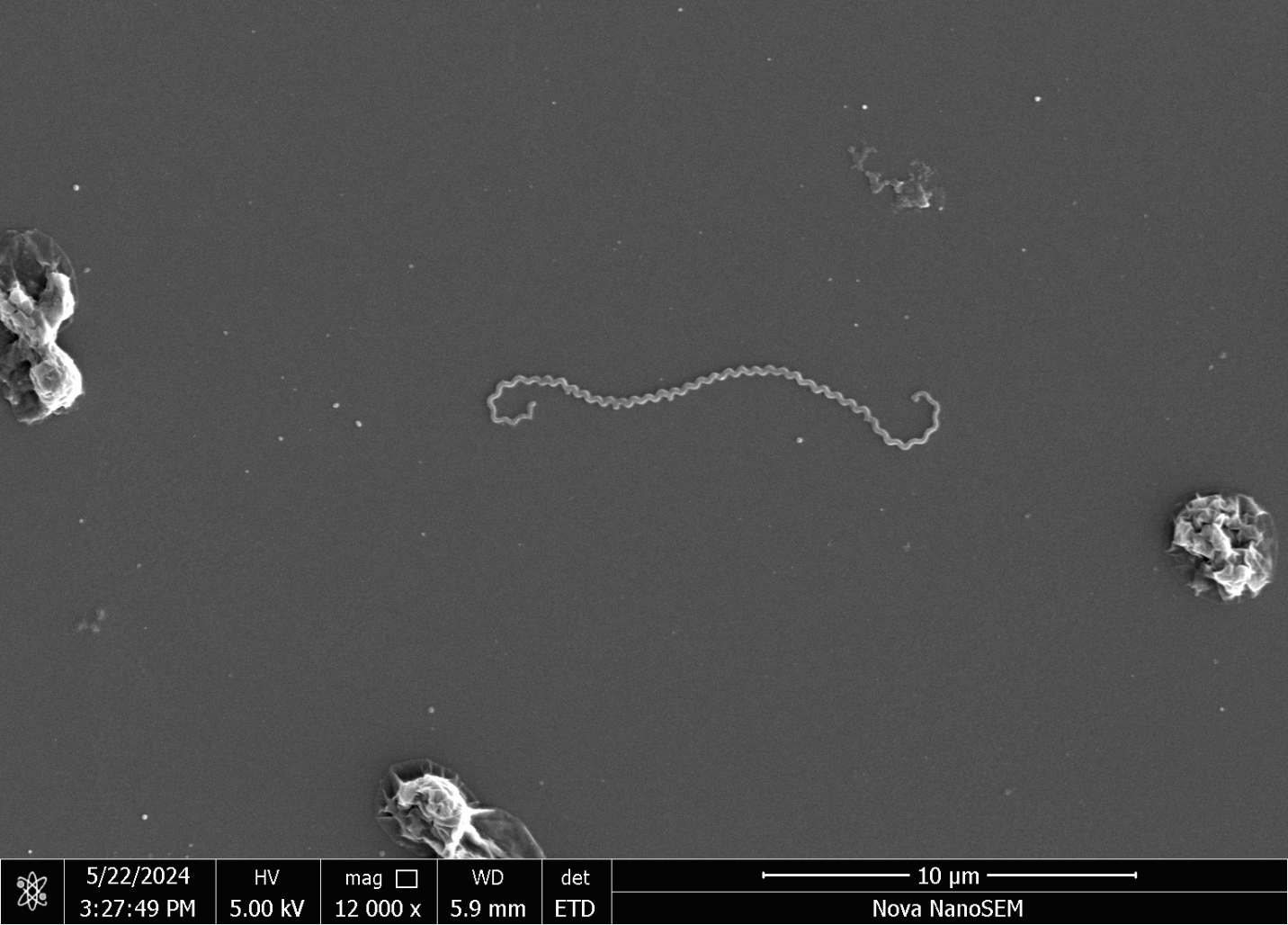


**Figure S6.** Scanning Electron Micrograph of a member of the Leptospiraceae family within a culture of *Nannochloropsis oceanica* IMET1.

**Text S2. Alpha and beta group significance**

Alpha diversity was analyzed using Faith’s Phylogenetic Diversity which incorporates phylogenetic relationships between the amplicon sequence variants (ASVs) and is unweighted. In general, there was no significant difference in alpha diversity between samples receiving no NaHCO_3_ (0 M) and 0.02 M NaHCO_3_ or between samples of different fraction sizes, 0.45 µm vs. 0.22 µm (Figures S7 and S8). The only samples that showed a significant difference in alpha diversity were Day 12 samples within the 0.22 µm fraction compared to Day 0 and Day 8 (p-value = 0.04). (Figure S9). Beta diversity was analyzed with Unifrac distances (unweighted). When comparing samples across timepoints (Day), regardless of size fractionation or addition of NaHCO_3_, samples from Day 12 had a significant difference in beta diversity when compared to Day 0, p-value= 0.04. (Figure S9). There was a significant difference in beta diversity between the 0.45 µm and 0.22 µm fraction, p-value= 0.006 (Figure S11). NaHCO_3_ input alone had no effect on beta diversity but did cause significant differences in beta diversity within the 0.22 µm fraction, p-value= 0.02 (Figures S12 and S13). There was only a significant difference (p-value = 0.013) between 0.45 µm-fractionated samples supplemented with 0 M NaHCO_3_ and 0.22 µm-fractionated samples supplemented with 0.02 M NaHCO_3_ which makes sense given the significant factors on beta diversity listed prior (Figure S13).


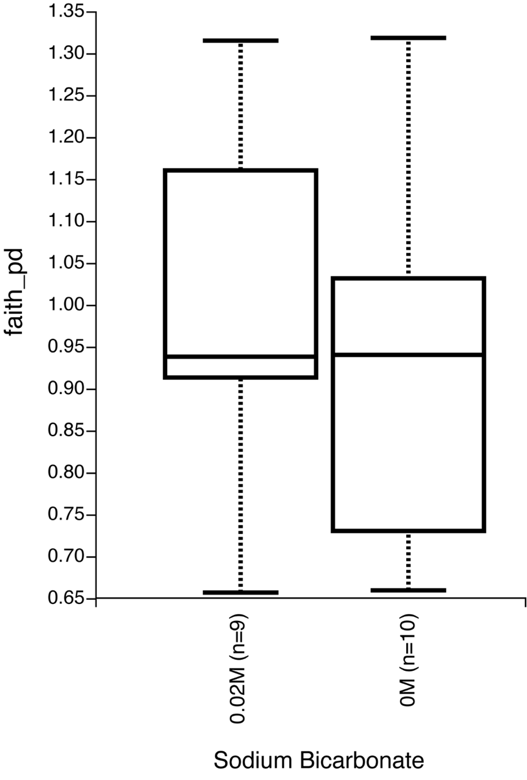


| Group 1 | Group 2 | H | p-value | q-value |
| --- | --- | --- | --- | --- |
| 0.02M (n=9) | 0M (n=10) | 0.32666667 | 0.5676285 | 0.5676285 |

**Figure S7.** Alpha group significance using Faith’s Phylogenetic comparing samples receiving no NaHCO_3_ (0 M) and 0.02 M NaHCO_3_.


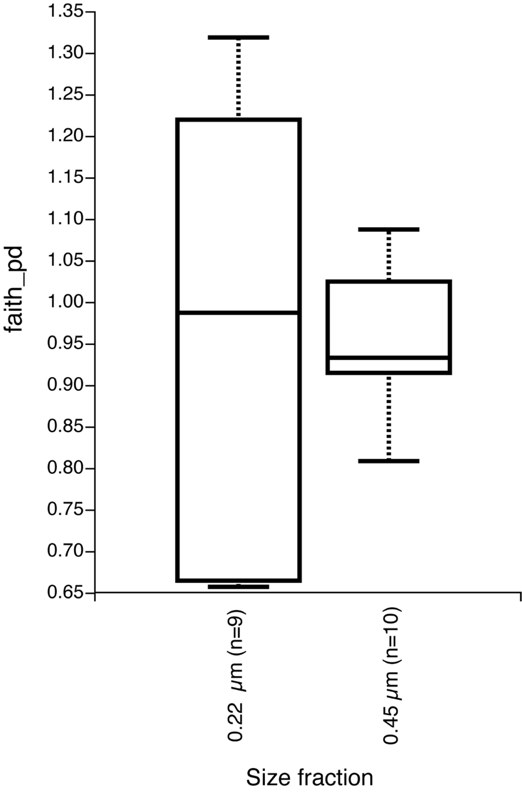


| Group 1 | Group 2 | H | p-value | q-value |
| --- | --- | --- | --- | --- |
| 0. 22 µm (n=9) | 0.45 µm (n=10) | 0.02666667 | 0.87028277 | 0.87028277 |

**Figure S8.** Alpha group significance using Faith’s Phylogenetic comparing samples of different fraction sizes, 0.45 µm vs. 0.22 µm.


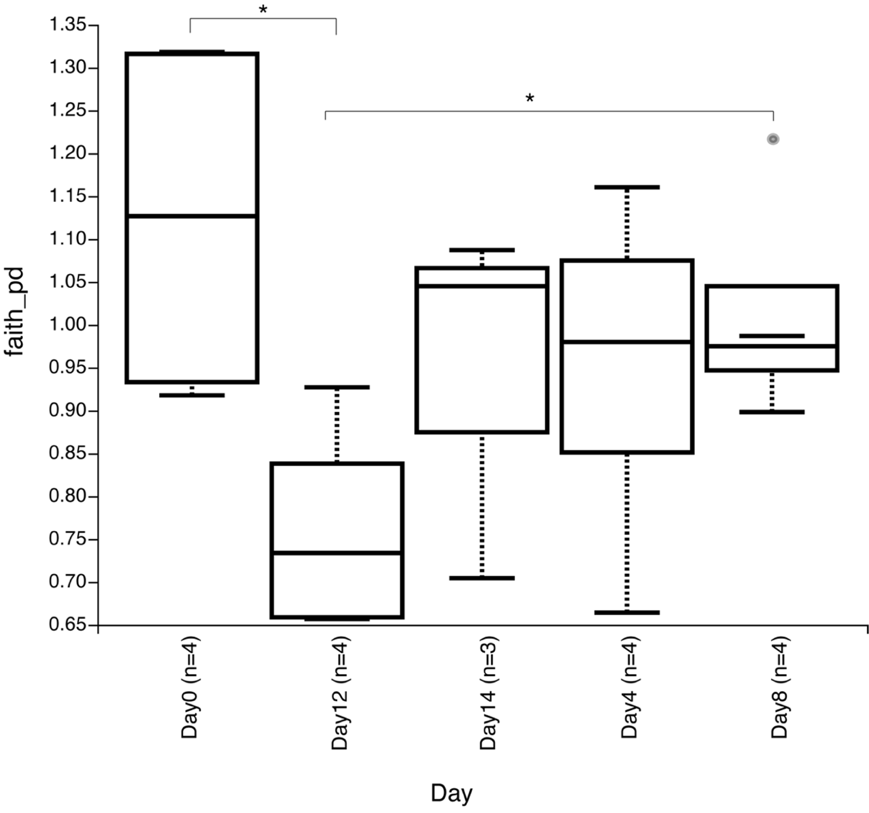


| Group 1 | Group 2 | H | p-value | q-value |
| --- | --- | --- | --- | --- |
| Day0 (n=4) | Day12 (n=4) | 4.08333333 | 0.04330814 | 0.21654071 |
| Day0 (n=4) | Day14 (n=3) | 0.5 | 0.47950012 | 0.79916687 |
| Day0 (n=4) | Day4 (n=4) | 1.33333333 | 0.24821308 | 0.49642616 |
| Day0 (n=4) | Day8 (n=4) | 0.33333333 | 0.56370286 | 0.8052898 |
| Day12 (n=4) | Day14 (n=3) | 2 | 0.15729921 | 0.39324802 |
| Day12 (n=4) | Day4 (n=4) | 2.08333333 | 0.14891467 | 0.39324802 |
| Day12 (n=4) | Day8 (n=4) | 4.08333333 | 0.04330814 | 0.21654071 |
| Day14 (n=3) | Day4 (n=4) | 0 | 1 | 1 |
| Day14 (n=3) | Day8 (n=4) | 0 | 1 | 1 |
| Day4 (n=4) | Day8 (n=4) | 0.08333333 | 0.77282999 | 0.96603749 |

**Figure S9.** Alpha group significance using Faith’s Phylogenetic comparing samples across timepoints (Day 0, 4, 8, 12, 14).


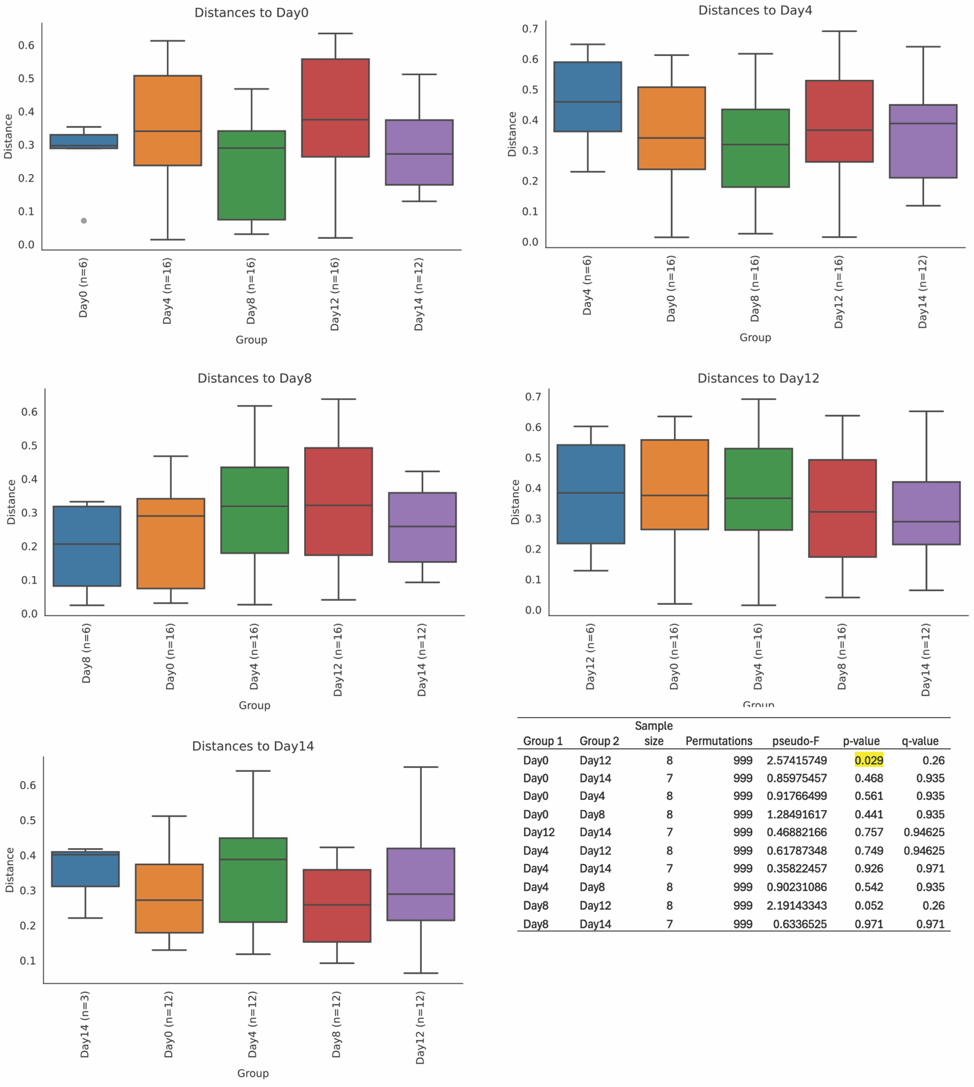


**Figure S10.** Beta diversity analyzed with Unifrac distances (unweighted) comparing samples across timepoints (Day 0, 4, 8, 12, 14).


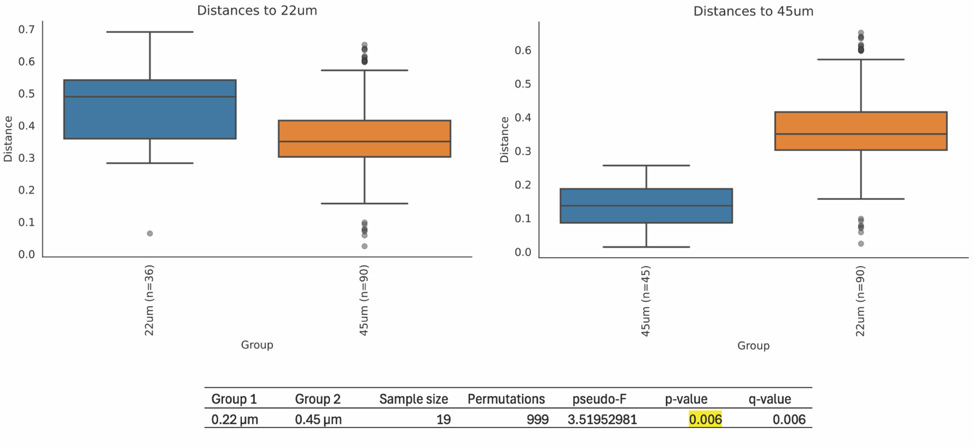


**Figure S11.** Beta diversity analyzed with Unifrac distances (unweighted) comparing samples of different fraction sizes, 0.45 µm vs. 0.22 µm.


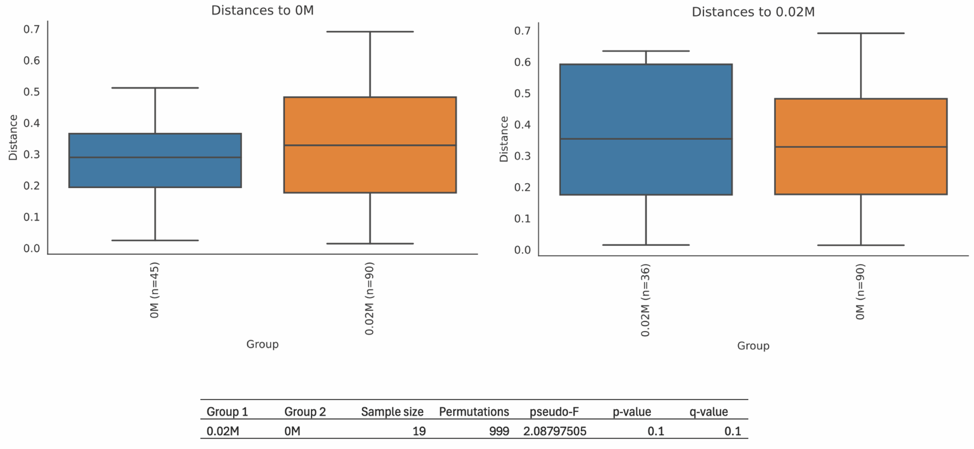


**Figure S12.** Beta diversity analyzed with Unifrac distances (unweighted) comparing samples receiving no NaHCO_3_ (0 M) and 0.02 M NaHCO_3._


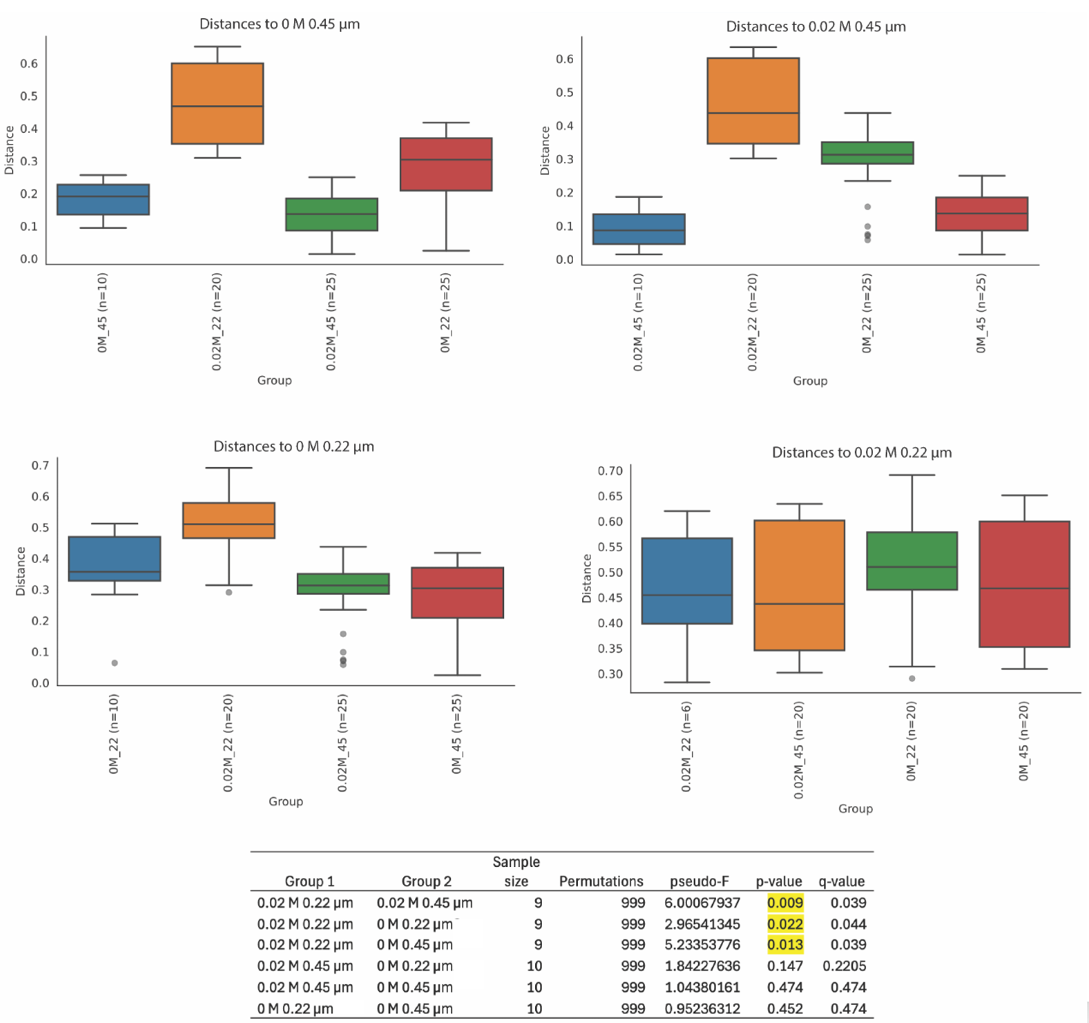


**Figure S13.** Beta diversity analyzed with Unifrac distances (unweighted) comparing 0.45 µm-fractionated samples supplemented with 0 M and 0.02 M NaHCO_3_ and 0.22 µm-fractionated samples supplemented with 0 M and 0.02 M NaHCO_3_.

**Text S3. Functional gene analysis through KEGG and PGPT annotation**

Figure S14 indicated that pathways related to Enzymes (ko01000) had the highest relative abundance in all three sequencing rounds. Predictably, other functional proteins such as Transporters (ko02000), DNA repair and recombination proteins (ko03400), and Ribosomes (ko03010) were present in each sequencing round in similar relative abundances. Figure 14C shows the specific parameters of these sequencing rounds. An additional relevant database for this study is the PLant-associated BActeria web resource (PLaBAse),^1^ which assigns a functional annotation of plant growth-promotion traits (PGPTs). PGPT Level 3 assignments (Figure 14B) are insightful for understanding the role of bacteria on carbon capture through microalgal growth enhancement. Relatively abundant PGPT functions with our system were Competitive Exclusion (CE) Bacterial Fitness, CE Bacteria Secretion, CE Cell Envelope Remodeling, CE Quorum Sensing Response, Colonization-Plant Cell Well, Colonization- Plant Derived Substrate Usage, Heavy Metal Detoxification, Neutralizing Abiotic Stress, Plant Vitamin Production, Phosphate and Potassium Solubilization, and Universal Stress Response. The PGPT annotation with the highest relative abundance in all three sequencing rounds was Plant Derived Substrate Usage (Figure 14B- blue bars). Relatively abundant PGPT functions with our system included Competitive Exclusion (CE) Bacterial Fitness, CE Bacteria Secretion, Heavy Metal Detoxification, Neutralizing Abiotic Stress, and Plant Vitamin Production etc. The PGPT annotation with the highest relative abundance was Plant Derived Substrate Usage (Figure S14B- blue bars). This is in line with the fact that some algae are known to release highly labile dissolved organic carbon (DOC) into the water that can then be consumed by nearby bacteria.^2^ PGPT Level3 Functions were found to be highly correlated (>0.5 correlation value) with various bacterial taxa found within our system. For example, Plant Vitamin Production genes had the highest correlation with Parcubacteria bacteria, *Gracilimonas* sp., Candidatus Kaiserbacteria bacteria, and Patescibacteria group bacteria, which suggest they may provide IMET1 with crucial vitamins needed for growth (Figure 8). Previous work found over half of surveyed microalgae require vitamin B_12_ through symbiotic bacteria for growth.^3^


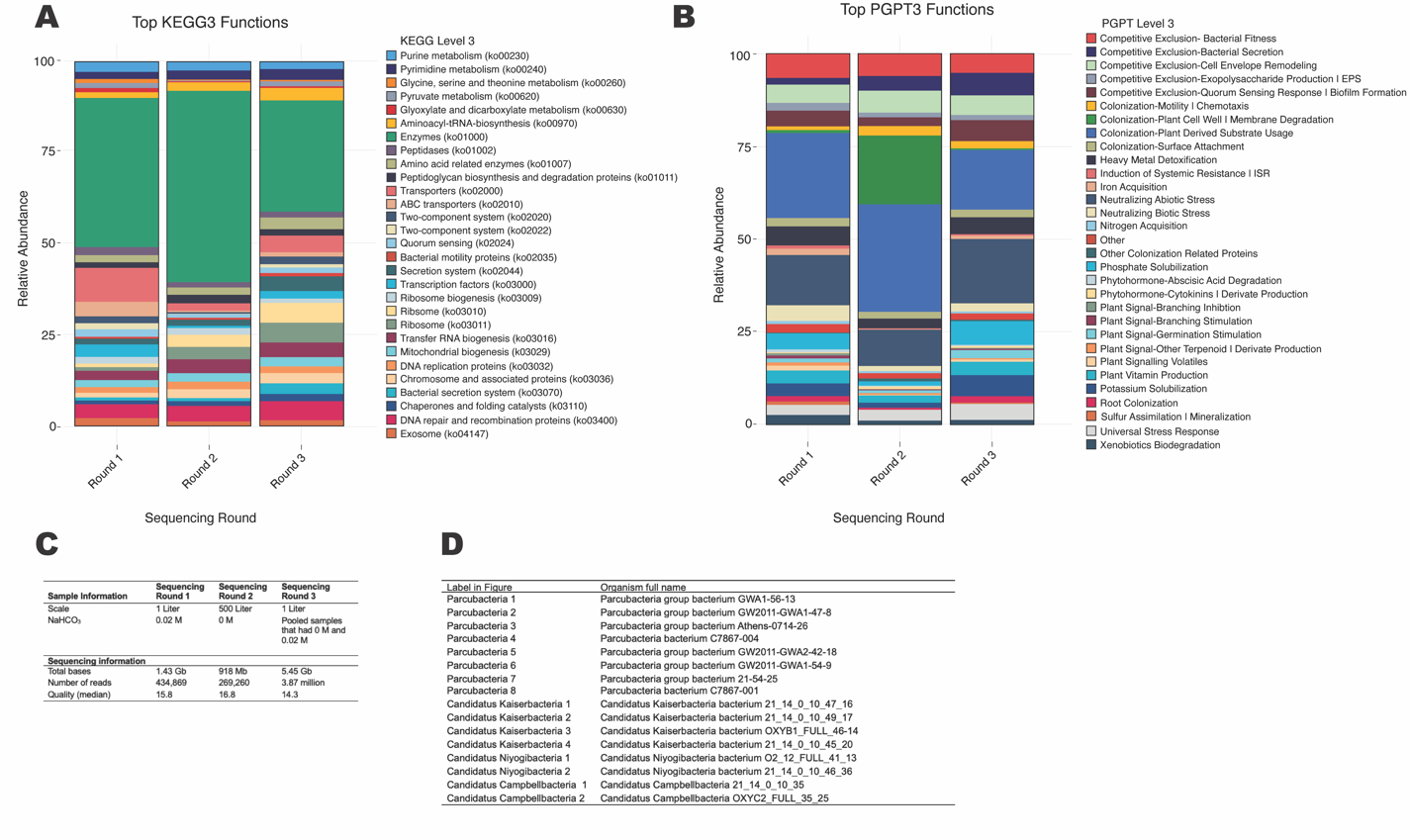


**Figure S14.**  Functional gene analysis from three rounds of metagenomic sequencing. (A) Stacked bar charts displaying the most abundant functions within KEGG Level 3, functions classified as “Other” have been removed. (B) Stacked bar charts displaying the most abundant functions within plant growth-promotion traits (PGPT) Level 3. (C) Table of experimental and sequencing details from the three rounds of metagenomic sequencing. (D) Table displaying the NCBI’s current full name of organisms abbreviated in heatmap, Figure 7 in manuscript.

**References**

(1) Patz, S.; Gautam, A.; Becker, M.; Ruppel, S.; Rodríguez-Palenzuela, P.; Huson, D. PLaBAse: A comprehensive web resource for analyzing the plant growth-promoting potential of plant-associated bacteria. *bioRxiv.* **2021**, (3).

(2) Larsson, U.; Hagström, A. Phytoplankton exudate release as an energy source for the growth of pelagic bacteria. *Mar. Biol.* **1979**, *52* (3). <https://doi.org/10.1007/BF00398133>.

(3) Croft, M.T.; Lawrence, A.D.; Raux-Deery, E.; Warren, M.J.; Smith, A.G. Algae acquire vitamin B12 through a symbiotic relationship with bacteria. *Nature* 2005, *438*, 90-93. <https://doi.org/10.1038/nature04056>
